# Qingfei mixture mitigates immunosuppression of tumor microenvironment in non-small cell lung cancer by blocking stat1/Ido1-mediated tryptophan-kynurenine pathway

**DOI:** 10.1016/j.heliyon.2024.e32260

**Published:** 2024-05-31

**Authors:** Zhuo Chen, Yu-Heng Ding, Lan Shao, Xu-Ming Ji, Xiang Qian, Ai-Qin Zhang

**Affiliations:** a Zhejiang Cancer Hospital, Hangzhou, Zhejiang, China; b School of Basic Medical Science, Zhejiang Chinese Medical University, Hangzhou, Zhejiang, China

**Keywords:** Lung cancer, Qingfei mixture, Tryptophan, Kynurenine, IDO1

## Abstract

Programmed death-1 (PD-1) acts as a T cell checkpoint and is important in controlling T cell exhaustion. Blocking the intercommunication across PD-1 and PD-L1 is promising for advanced lung cancer treatment. However, the response rate requires being strengthened. This study aimed to determine whether the combination treatment of Qingfei mixture (QFM) and PD-1 inhibitor could improve the sensitivity of monoclonal antibody by regulating STAT1/IDO1-mediated tryptophan (Trp)-kynurenine (Kyn) pathway. The *in vivo* imaging system, immunofluorescence, hematoxylin-eosin staining, TUNEL, flow cytometry, HPLC, and ELISA were used to estimate the anti-tumor effects in LLC-luc tumor-bearing C57BL/6 mice treated with QFM, PD-1 inhibitor, 2-NP (enhancer of STAT1 transcription), and FICZ (AhR agonist) alone or in combination. IFN-γ-mediated A549 and LLC cells were treated with QFM-containing serum and fludarabine (FLU, STAT1 inhibitor), and cell viability, apoptosis, and Kyn content were then evaluated using CCK-8 assays, flow cytometry, and HPLC assays, respectively. Additionally, the expressions of STAT1, IDO1, AhR, NFATc1, TRIP12, PD-1, and PD-L1 were measured *in vivo* and *in vitro*. We found QFM increased the anti-cancer actions of PD-1 inhibitors by increasing the CD8*+*IFNγ^+^ T cells infiltration and decreasing the ratio of Kyn/Trp. Besides, QFM-containing serum suppressed the proliferation and promoted apoptosis in A549 and LLC cells, meanwhile, FLU boosted the effects of QFM-containing serum. Moreover, the suppression of tumor growth in the combination therapy was attenuated in the mice receiving 2-NP or FICZ. The occurrence of the above results was accompanied by a decrease in STAT1, IDO1, AhR, PD-1, and PD-L1 expressions. Collectively, the findings suggested that QFM may increase the influences of PD-1 inhibitors at least partially by blocking the STAT1/IDO1-mediated tryptophan-kynurenine pathway in lung cancer.

## 1 Introduction

Lung cancer (LC) is one of the most lethal malignancies, of which non-small cell lung cancer (NSCLC) accounts for about 80 %, and most of the patients are already in the middle to late stage at the time of diagnosis and then are unable to be managed surgically [1]. Platinum-based combination chemotherapy is the cornerstone of treatment for advanced patients, and the survival benefit has reached a bottleneck [2]. Despite the extraordinary success of molecularly targeted medications, no effective solution has been found for driver mutation-negative (approximately 70 % of patients) or drug-resistant patients [3]. In current times, the development of immune checkpoint inhibitors (ICIs) has remolded the landscape of clinical intervention of tumors, with a significant increase in 5-year survival, and the most widely used of them are PD-1/PD-L1 immune-checkpoint inhibitors [[4], [5], [6]]. However, PD-1 inhibitors have low efficacy in monotherapy, and high doses are prone to toxic side effects such as immunopneumonia, cough, rash, and cardiovascular toxicity [7,8]. Hence, more effective treatment modalities are urgently needed in the clinic.

Traditional Chinese medicine (TCM) has taken an active position in the clinical practice of tumor prevention and treatment, and its multi-pathway and multi-targeted intervention features have been effective in increasing the efficacy and reducing the toxicity in the comprehensive treatment of lung cancer [9,10]. Combination therapy of PD-1 inhibitors has been widely utilized in the management of human cancers, including NSCLC. For example, Zhang et al. reported that a PD-1 inhibitor combined with anlotinib, an anti-angiogenic drug, could effectively improve disease control rate and prolong progression-free survival in NSCLC patients [11]. Additionally, Liu et al. found that nursing intervention combined with PD-1 inhibitor significantly ameliorated the general state of life of patients with lung cancer after chemotherapy [12]. More importantly, another study demonstrated that ICIs alone minimize the risk of fatal adverse events, while conjugated strategies with chemotherapy have the reverse effect when compared to standard chemotherapy [13]. However, clinical studies have not yet seen a systematic and in-depth mechanism interpretation. Hence, it is of great scientific significance and clinical value to deeply inquire into the connotation of TCM in the immunotherapy of PD-1 inhibitors for lung cancer to increase efficacy and reduce toxicity.

Qingfei mixture (QFM) is a prescription that has been therapeutically employed as a hospital preparation for more than 40 years and has been widely recognized by the clinic in Zhejiang Cancer Hospital (Zhejiang, China) due to its good efficacy, good safety, and affordable price. Previous clinical studies have confirmed that QFM improves immune function and therapeutic effect in patients with advanced lung cancer [14,15]. It also increases the abundance of beneficial intestinal bacteria and promotes the release of anti-tumor immune factors in lung cancer mice [16]. However, the exact mechanism of QFM in the treatment of lung cancer is currently less studied, especially in the combination of PD-1 inhibitors.

Recently, the deepening understanding of the relationship between tumor immune microenvironment (TIME) and drug resistance mechanisms has provided new perspectives for the interpretation of the anti-tumor mechanism of TCM. Focusing on the effects of TCM on the TIME may be one of the important directions to improve the immunotherapy of lung cancer. PD-1 expression of T-cells is influenced by several variables, including the strength of TCR signaling, ubiquitylation modification, activation of tumor signaling pathways, cytokines, etc [[17], [18], [19]]. Recent studies have shown that abnormal tryptophan metabolism in the TIME is one of the main mechanisms of elevated PD-1 expression in CD8^+^ T cells [20,21]. Indoleamine 2,3-dioxygenase 1 (IDO1), as a rate-limited enzyme necessary for catalytic tryptophan metabolism, is highly expressed in lung cancer cells, converting more than 95 % of tryptophan (Try) to kynurenine (Kyn), leading to tryptophan depletion and the accumulation of downstream products, then forming an immunosuppressive microenvironment [22,23]. A clinical study has shown that effector T cells are activated in the patient's organism under the action of IDO inhibitors, and combined ICIs could inhibit tumor growth more effectively than single-agent ICIs [24]. It is suggested that IDO1 is an important target for regulating tryptophan metabolism in the lung cancer microenvironment to improve the efficacy of PD-1 inhibitors. On the other side, CD8^+^ T cells release the inflammatory factor IFN-γ, which induces the phosphorylation of STAT1 by transmitting signals into tumor cells through its receptor in the TIME. Phosphorylated STAT1 enters the nucleus as a homodimer and initiates the expression of IDO1 [25]. Moreover, we previously found that STAT1 is one of the core targets of QFM against lung cancer according to network pharmacology and bioinformatics analyses. Therefore, we hypothesized that QFM may inhibit IDO1 for improving tryptophan metabolism by down-regulating STAT1 phosphorylation to decrease PD-1 expression in CD8^+^ T cells in lung cancer.

In the present investigation, we looked at the effects of QFM combined with PD-1 inhibitor on tryptophan metabolic reprogramming pathway *in vitro* and *in vivo*, which provided a novel route for the synergistic immunotherapy research of QFM in lung cancer.

## 2 Method and materials

### 2.1 Preparation of QFM

The crude drugs of QFM were purchased from the pharmacy of Zhejiang Cancer Hospital and authenticated by Professor Ai-Qin Zhang (Zhejiang Cancer Hospital, China). A total of 204.6 g raw QFM was put into a clean porcelain jar and soaked for 30 min [26]. Ten times of distilled water was added into the container and boiled twice for 2 h each. All the supernatants were harvested and concentrated to 2.66 g/ml in the induction cooker and stored at 4 °C.

### 2.2 Cell culture

Human lung carcinoma A549 cells, Lewis lung cancer (LLC) cells, and murine Lewis lung carcinoma luciferase (LLC-Luc) cells were acquired from iCell Bioscience Inc (Shanghai, China). All cells were kept in DMEM (TRANSGEN BIOTECH, Beijing, China) complemented with 10 % FBS (GIBCO) in the cell incubator with 5 % carbon dioxide.

### 2.3 Bioluminescence imaging

Male C57BL/6 mice (Aged 6–8 weeks) were acquired from the SLAC. LTD (Shanghai, China) and placed in an SPF environment. Mice were injected with 100 μl LLC-luc cells suspension (1 × 10^7^ cells/mL) through the tail veins. After one week, the animals were separated and randomized into 4 groups as follows: model, QFM, anti-PD-1, and QFM + anti-PD-1 groups with n = 6 in each group. Mice in the model group were treated with 0.4 mL purified water. Mice in the QFM group were orally administered 0.4 mL QFM. Mice in the anti-PD-1 group were managed with 200 μg PD-1 inhibitor [BE0146, InVivoMAb anti-mouse PD-1 (CD279), Bioxcell, USA) every other day via intraperitoneal injection. In the QFM + anti-PD-1 group, the mice accepted intraperitoneal injections of 200 μg anti-PD-1 inhibitor every other day and intragastrically administrated with 0.2 mL QFM every day. After 14 days of continuous treatment, bioluminescence was detected by imaging mice with 15 mg/mL D-luciferin (10 μL/g body weight, #ST196, Beyotime, China). At the end of the research, all mice were devoted and the lung tumors were harvested for further analysis.

### 2.4 Immunofluorescence staining

Lung tumor tissues of mice were removed on ice and fixed in 4 % paraformaldehyde for over 24 h. After that, the immunofluorescence staining was conducted on tumor paraffin sections with 4 μm thickness. After antigen retrieval and blocked in 5 % serum, the sections were incubated with PD-1 (ab214421, Abcam) and CD8 (sc-18913, Santa) at 4 °C for an entire night. The sections were then treated with matching fluorescence-conjugated secondary antibodies for 60 min. Next, the nuclei were counterstained with DAPI (ab104139, Abcam). Finally, all sections were mounted with neutral gum and photographed.

### 2.5 Preparation of QFM-containing rat sera

Male SD rats were provided by the SLAC and were independently separated into two groups (n = 6/group): the control and the QFM-containing serum groups. A dose of QFM equal to 26.6 g/kg of body weight was given intragastrically to rats in the QFM-containing serum group daily for one week [26]. Rats in the normal group had an equal volume of distilled water as QFM. At last, blood was extracted from the abdominal aortas of rats. The serum was then collected by centrifuging at 3000 rpm for 15 min and inactivated at 56 °C, and subsequently filtered by a 0.22 μm membrane. The serum containing the QFM was finally stored at −80 °C.

### 2.6 CCK-8 assay

A549 and LLC cells were cultured with IFN-γ (100 ng/ml, Beijing Solarbio Science & Technology Co., Ltd, China), 10 % QFM-containing serum, and fludarabine (FLU, 5 μM, STAT1 inhibitor, MedChemExpress) alone or in combination for 24 h supplemented with exogenous Try. After incubation, CCK-8 reagent (Beyotime, China) was put into each well and prepared for 3 h in the shadows. Finally, the absorbance was determined by using a microplate reader (450 nm, Molecular Devices, USA).

### 2.7 Cellular apoptosis assay

Cellular apoptosis in the A549 and LLC cells treated with IFN-γ, QFM-containing serum, and the FLU alone or in combination was detected in this study. After treatment with test substances, the cells underwent incubation with 5 μL of Annexin V-FITC and 10 μL of PI for 15 min. Next, the apoptosis levels of cells were examined by a flow cytometer (BD Biosciences).

### 2.8 Animal studies

Another cohort of male C57BL/6 mice was also injected with 0.1 ml of the cell suspension (1 × 10^7^ cells/ml) into the tail veins. Around one week following injection, the mice were equally split into six teams. Mice in the six groups were governed normal water (Control and model groups), PD-1 inhibitor [Anti-PD-1; 200 μg of PD-1 inhibitor, intraperitoneally (i.p.), every other day (qod)], PD-1 inhibitor + QFM (Anti-PD-1+QFM; 200 μg of PD-1 inhibitor, qod+0.4 ml of QFM, i.g., daily), PD-1 inhibitor + QFM + enhancer of STAT1 transcription (Anti-PD-1+QFM+2-NP; 200 μg of PD-1 inhibitor, i.p., qod+0.4 ml of QFM, i.g., daily+10 mg/mL of 2-NP, i.p., qod), and PD-1 inhibitor + QFM + AhR agonist (Anti-PD-1+QFM + FICZ; 200 μg of PD-1 inhibitor, i.p., qod+0.4 ml of QFM, i.g., daily+10 mg/mL of FICZ, i.p., qod), respectively, for 14 days. Subsequently, the bioluminescence was also detected by imaging mice as previously described. After the last treatment on day 14, the blood samples were collected and centrifuged to obtain serum for ELISA. Additionally, the lung tumor tissues were extracted, photoed, fixed, and then embedded in paraffin for H&E staining and TUNEL assays, while the other part of the tumor tissues was used for CD8^＋^IFN-γ^+^ T cells analysis and stored at −80 °C for high-performance liquid chromatography (HPLC) and western blotting analysis. Moreover, the thymus and spleen tissues were extracted and weighted to calculate the thymus index and spleen index, respectively.

### 2.9 Flow cytometry analysis of CD8^＋^ and CD8^＋^IFN-γ^+^ T cells

Tumor-infiltrating lymphocytes were isolated from the lung tumor tissues. Cells were stained with anti-mouse-CD8 (sc-18913) fluorescent antibody to determine the percentage of positive cells. The frequency of CD8^+^IFN-γ^+^ T cells was measured with intracellular cytokine staining using an anti–IFN–γ antibody (#560660, BD Biosciences). Finally, the stained cells were analyzed by the flow cytometer.

### 2.10 ELISA analysis

The tumor tissues were completely ground using a homogenizer and centrifuged. Subsequently, the TNF-α and IFN-γ levels in tumor tissues, the levels of Kyn in the culture supernatant of treated A549 and LLC cells, and IL-2, TNF-α, and IFN-γ in the serum were determined using commercial ELISA kits.

### 2.11 HPLC analysis of Trp and Kyn

Trp and Kyn concentrations in lung tumor tissues were tested by HPLC. Briefly speaking, lung cancer tissues were extracted from each group of mice, and 1 ml of cold methanol was added and broken by ultrasonication on ice. The supernatant was extracted and centrifuged at 12,000 rpm for 10 min. The samples were separated on an ExionLCAD Ultra Performance Liquid Chromatography (UHPLC) ACQUITY UPLC HSS T 3 column (100 mm × 2.1 mm, 1.8 μm). The temperature of the injection plate was 4 °C. The temperature of the column was 40 °C, and the injection volume was 4 μL [27].

### 2.12 Histology examination and TUNEL analysis

After the *in vivo* experiments, the fixed lung tumors were cut into 4 μm slices. Then, the sections were stained with hematoxylin and eosin to assess histopathological changes. Besides, the TUNEL assay was performed to detect the cell apoptosis rate of lung tumor tissues. The nucleus was counterstained with DAPI. The TUNEL-positive cells were observed using a microscope (Nikon).

### 2.13 qPCR analysis

Total RNA was obtained from lung tumor tissues using TRIzol reagent (Invitrogen) and then reverse transcribed into cDNA. qPCR was conducted with SYBR Green Master mix on a Real-Time PCR System (Roche, Switzerland). The sequences for primers were synthesized by Sangon (Shanghai, China) as follows: AhR forward primer 5′-TAAACGACACAGAGACCGGC-3′ and reverse primer 5′-TCCCTGTAGAAAGCCCTTACC-3'; NFATc1 forward primer 5′-ATGCCAAGTACCAGCTTTCCA-3′ and reverse primer 5′-GGTCTCGGCAATTCCTGCAT-3'; GAPDH forward primer 5′-AAGGTCGGTGTGAACGGATTT-3′ and reverse primer 5′-CTTTGTCACAAGAGAAGGCAGC-3'. The levels of AhR and NFATc1 were analyzed utilizing the 2^–ΔΔCT^ approach, with GAPDH serving as the endogenous control.

### 2.14 Western blot

Total protein was produced from treated A549 and LLC cells and frozen lung tumor tissues by RIPA Lysis Buffer (Beyotime, China) with protease inhibitor (CW2200S, JiangSu CoWin Biotech Co., Ltd. China). The concentration of protein samples from tissue homogenates and cell lysates was measured with a BCA assay (pc0020, Beyotime). Equal amounts of protein were isolated by SDS-PAGE and then transferred to PVDF membranes. After that, the membranes were treated with 5 % non-fat powdered milk for 1 h and then incubated with the primary antibodies: Phospho-STAT1 (AF3299, 1:1000, Affinity), anti-STAT1 (9172S, 1:1000, CST), anti-IDO1 (DF6502, 1:1000, Affinity), anti-AhR (ab309491, 1:1000, Abcam), anti-NFATc1 (ab25916, 1:1000, Abcam), anti-TRIP12 (ab313628, 1:1000, Abcam), anti-Bax (ab32503, 1:1000, Abcam), anti-Bcl-2 (ab182858, 1:1000, Abcam), anti-Cleaved caspase-3 (ab214430, 1:1000, Abcam), anti-PD-1 (ab214421, 1:1000, Abcam), anti-PD-L1 (ab213480, 1:1000, Abcam), and anti-GAPDH (10494-1-AP, 1:5000, proteintech) overnight at 4 °C. Afterward, the membranes were then incubated with the appropriate secondary antibody. Finally, the bands were visualized using chemiluminescence and quantified by Image J software (Bethesda, MD).

### 2.15 Statistical analysis

All results were presented as the means ± standard deviation. Comparison within the two groups was carried out using a student's unpaired *t*-test. Difference among multi-groups was compared by using the ANOVA. *P<0.05* was considered statistical significance.

## 3 Results

### 3.1 QFM combined with PD-1 inhibitor hindered tumor growth *in vivo*

**Fig. 1:**
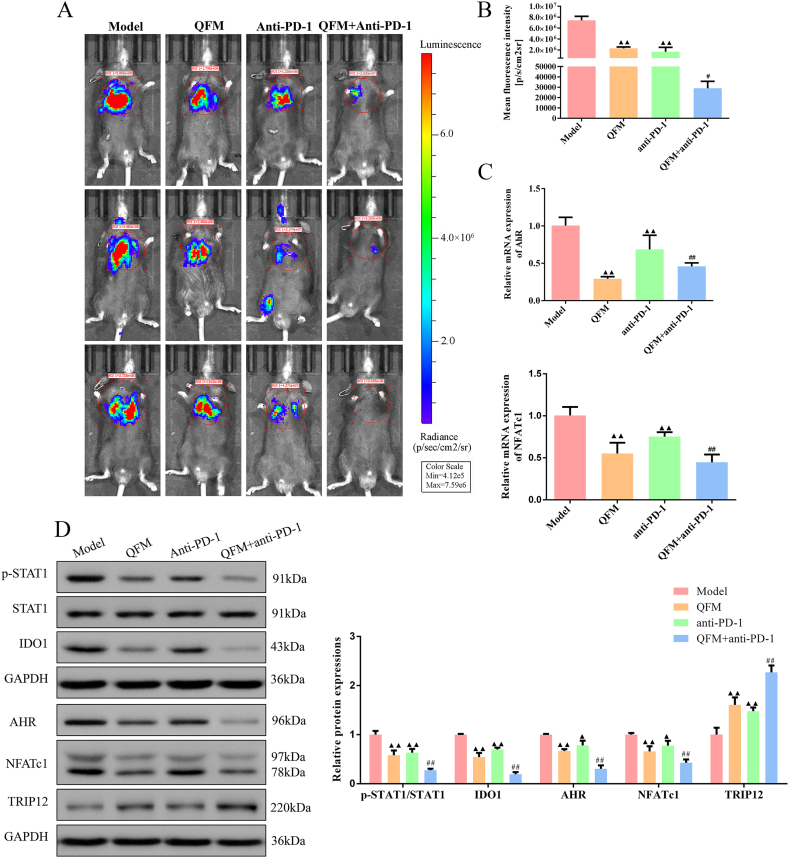
**Effect of QFM combined with PD-1 inhibitor on tumor growth in LC mice *in vivo* by targeting STAT1/IDO1-Trp-Kyn pathway.** (A) Representative images with fluorescence signal in living LC mice. (B) The mean of fluorescence signal in LC mice. (C) The mRNA level of AhR and NFATc1 in tumor tissues of LC mice using quantitative RT-PCR analysis. (D) Western blot measured the expression of p-STAT1, STAT1, IDO1, AhR, NFATc1, and TRIP12 in tumor tissues of LC mice (The original image is provided in the Supplementary file Fig. S1). ^▲^*P < 0.05*, ^▲▲^*P < 0.01* compared with model group; ^#^*P < 0.05*, ^##^*P < 0.01* compared with anti-PD-1 group.

Living fluorescence imaging was first used to observe the tumor growth in mice. As illustrated in Fig. 1A-B, the mean flux of mice was significantly decreased after QFM and PD-1 inhibitor treatment. In particular, when QFM and PD-1 inhibitors were combined, the difference between the mice in the QFM + anti-PD-1 group and the anti-PD-1 group alone was enhanced. A study by Liu et al. reported that tumor-repopulating cells promoted PD-1 up-regulation in CD8^+^ T cells through activation of the Kyn-AhR pathway [28]. Also, Qin et al. found that adequate tryptophan in T cells enhances the degradation of NFATc1 by tryptophanylated modification of TRIP12 through tryptophanyl-tRNA synthetase (WARS), which reduces PD-1 levels on the surface of T cells [20]. To evaluate the mRNA expression levels of AhR and NFATc1 in mice, RT-qPCR assays were carried out. As shown in Fig. 1C, AhR, and NFATc1 mRNA levels were observably lower in the QFM group and QFM + anti-PD-1 group than in the model and anti-PD-1 groups, respectively. Furthermore, the protein expression levels of p-STAT1, IDO1, AhR, as well as NFATc1, were significantly downregulated in treated groups, particularly in the QFM + anti-PD-1 group. Interestingly, QFM and anti-PD-1 treatment alone or in combination could increase TRIP12 expression in lung tumors of LLC-luc-bearing mice (Fig. 1D; Fig. S1). These results indicated that QFM and PD-1 inhibitors coordinately attenuated tumor growth in mice and were mitigated by inhibition of the STAT1/IDO1 pathway.

### 3.2 QFM combined with PD-1 inhibitor attenuated the KYN/TRP ratio and promoted proinflammatory cytokines levels

**Fig. 2:**
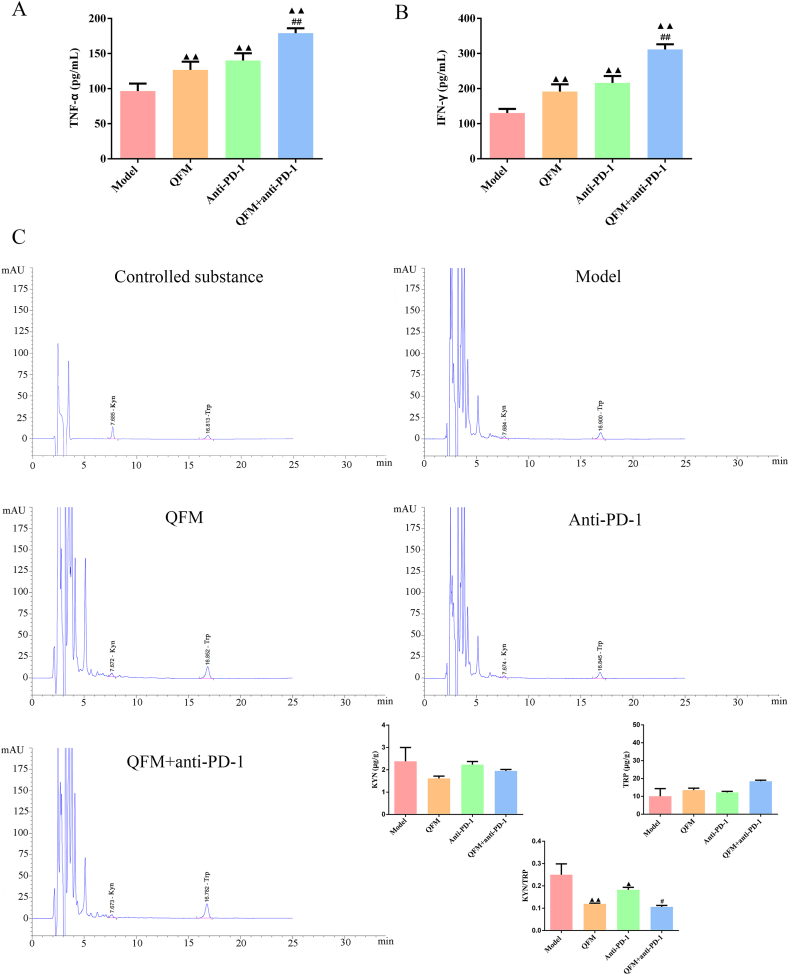
**Effect of QFM combined with PD-1 inhibitor on TNF-α, IFN-γ, Trp, and Kyn contents in LC mice.** (A) TNF-α and (B) IFN-γ levels in the tumor tissues of LC mice were detected by using ELISA. (C) Trp and Kyn contents in the tumor tissues of each group of LC mice were assessed using HPLC. ^▲▲^*P < 0.01* compared with the model group; ^##^*P < 0.01* compared with the anti-PD-1 group.

With regard to literature, TNF-α is often employed in tumor biotherapy, whereas IFN-γ has been shown to mitigate tumor cell proliferation [29,30]. In this investigation, we found that QFM and anti-PD-1 treatment alone or in combination significantly increased the TNF-α and IFN-γ levels in lung tumors of mice (Fig. 2A-B). Moreover, we also measured the concentrations of Trp and Kyn by HPLC. The data suggest that QFM and anti-PD-1 treatment alone or in combination significantly reduced the Kyn/Trp ratios in the lung tumor of mice, while there were no distinct differences in Kyn and Trp levels in LLC-luc bearing mice (Fig. 2C). These results suggested that the QFM coupled with the PD-1 inhibitor ameliorated the immunosuppressive microenvironment via the tryptophan-kynurenine pathway.

### 3.3 QFM coupled with PD-1 inhibitor induced infiltration of CD8^+^ T-cells in LLC-luc bearing mice

**Fig. 3:**
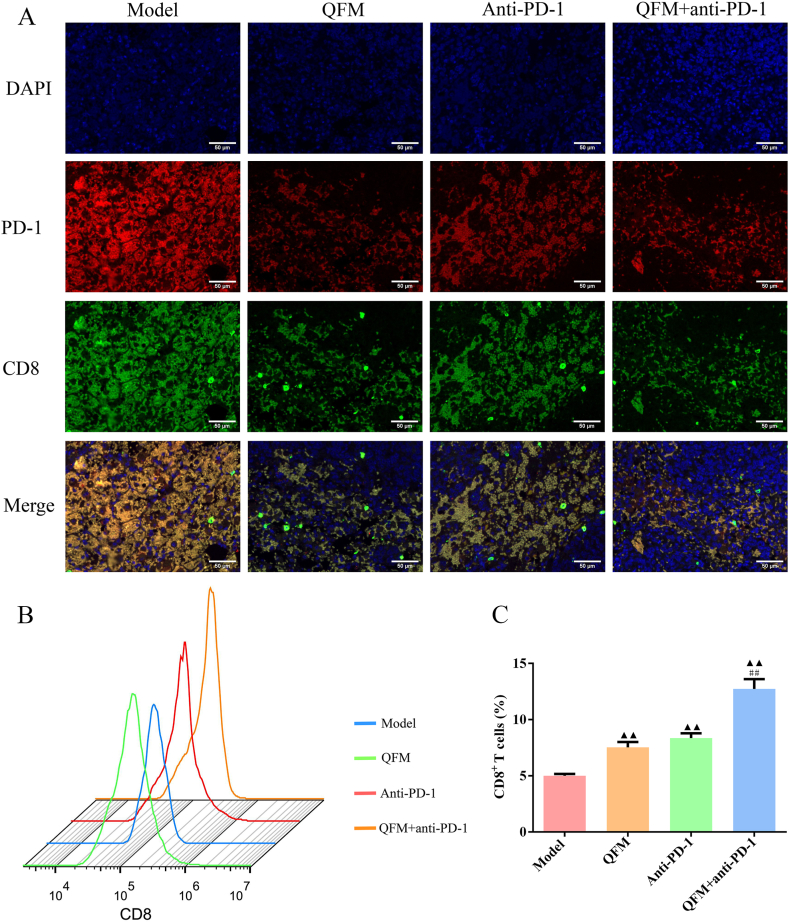
**Effect of QFM combined with PD-1 inhibitor on tumor-infiltrating CD8**^**+**^**T-cells and PD-1 expression in LC mice.** (A) The immunofluorescence detected the effect of QFM combined with PD-1 inhibitor on the expression of PD-1 and IDO1 in tumor tissues of LC mice (Scale bar = 50 μm). (B, C) Flow cytometry detected the percentage of CD8^+^ T cells in tumor tissues of LC mice. ^▲^*P < 0.05*, ^▲▲^*P < 0.01* compared with model group; ^##^*P < 0.01* compared with anti-PD-1 group.

Next, we analyzed the impacts of QFM and anti-PD-1 treatment alone or in combination in the lung tumor tissues of mice by immunofluorescence staining and flow cytometry analysis of CD8^+^ cells. We observed that the populations of CD8^+^ T-cells in tumor tissues from QFM and anti-PD-1 alone or in combination-treated mice were significantly increased compared to those treated with physiological saline, as accompanied by a decrease of PD-1 expression levels (Fig. 3A-B). Collectively, these results suggested that QFM coupled with PD-1 inhibitor might affect the cellular functions of CD8^+^ T cells via STAT1/IDO1-mediated Trp-Kyn metabolism.

### 3.4 QFM repressed cell proliferation and promoted apoptosis of LC cells via STAT1/Ido1 pathway

**Fig. 4:**
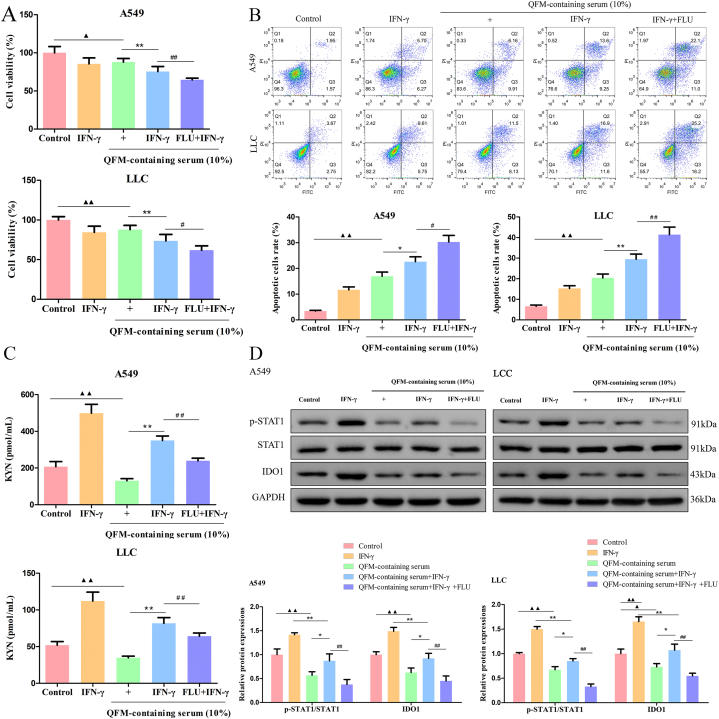
**Effect of QFM-containing serum on STAT1 to inhibit IDO1-Trp-Kyn signaling pathway *in vitro*.** (A) Cell Counting Kit-8 (CCK-8) was used to detect the cell viability of IFN-γ-mediated A549 and LLC cells treated with QFM-containing serum or in combination with FLU (5 μM, STAT1 inhibitor). (B) Flow cytometric analysis on the cell apoptosis in IFN-γ-mediated A549 and LLC cells. (C) ELISA for detecting Kyn contents in IFN-γ-mediated A549 and LLC cells. (D) Western blotting for detecting p-STAT1, STAT1, and IDO1 expression levels in IFN-γ-mediated A549 and LLC cells (The original image is provided in the Supplementary file Fig. S2). ^▲^*P < 0.05,*^▲▲^*P < 0.01* compared with control group; *P<0.05, **P<0.01 compared with QFM-containing serum group; ^#^*P < 0.05,*^##^*P < 0.01* compared with QFM-containing serum + IFN-γ group.

In the immune microenvironment of lung cancer, IFN-γ mediates the phosphorylation of STAT1 and promotes the expression of IDO1 in LC cells. IDO1, as an important negative immune regulatory molecule in LC, enhances the depletion of Trp and promotes the synthesis of Kyn, which leads to the rise of PD-1 levels in CD8^+^ T cells and the formation of immune escape. The impact of QFM-containing serum on cell proliferation, apoptosis, Kyn metabolism, and STAT1/IDO1 pathway was next investigated. The results found that QFM-containing serum significantly decreased the viability of IFN-γ-induced A549 and LLC cells (Fig. 4A). Also, the apoptosis rate of IFN-γ-induced A549 and LLC cells was increased after treatment with QFM-containing serum for 24 h (Fig. 4B). Especially, our result showed that treatment with QFM-containing serum decreased the concentration of Kyn in IFN-γ-induced A549 and LLC cells (Fig. 4C). We further investigated the effect of QFM-containing serum treatment on the STAT1/IDO1 pathway. Exposure of IFN-γ-induced A549 and LLC cells to QFM-containing serum resulted in diminished levels of the phosphorylated form of STAT1, as well as IDO1 levels (Fig. 4D; Fig. S2). Notably, the effects of QFM-containing serum in IFN-γ-induced A549 and LLC cells were significantly enhanced when also exposed to FLU (Fig. 4A-D). These results revealed that QFM-containing serum triggered A549 and LLC cells apoptosis *in vitro* via STAT1/IDO1 pathway.

### 3.5 2-NP or FICZ attenuated the inhibition of QFM coupled with PD-1 inhibitor *in vivo*

**Fig. 5:**
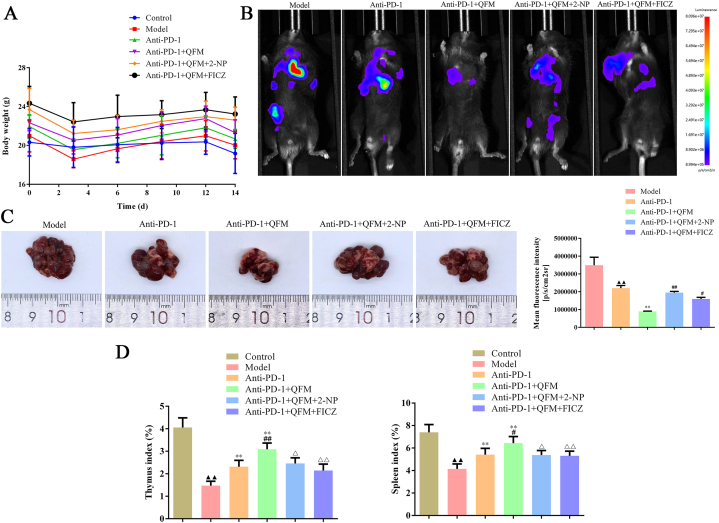
**Effect of 2-NP or FICZ on the anticancer activity of QFM combined with a PD-1 inhibitor in LC mice *in vivo.*** (A) Body weight change for LC mice in each group. (B) Representative images with fluorescence signal in LC mice treated with QFM and PD-1 inhibitor combined with 2-NP or FICZ. (C) Representative images of tumor tissues in the lung of LC mice. (D) Thymus and spleen index of LC mice in each group. ^▲▲^*P < 0.01* compared with control group; **P<0.01 compared with model group; ^#^*P < 0.05,*^##^*P < 0.01* anti-PD-1group; ^△^*P < 0.05,*^△△^*P < 0.01* anti-PD-1+QFM group.

To further identify the role of STAT1 and AhR on the role of QFM coupled with a PD-1 inhibitor, the rescued experiments were performed in LLC-luc-bearing C57BL/6 mice. As shown in Fig. 5A, there were no significant differences in body weight that showed a similar tendency. Also, anti-PD-1, and anti-PD-1+QFM treatment significantly decreased the mean fluorescence intensity of mice bearing LLC-luc tumor (Fig. 5B-C). Similarly, the thymus index and spleen index were elevated by anti-PD-1 and anti-PD-1+QFM treatment, however, the effects of anti-PD-1+QFM on tumor growth, thymus index and spleen index can be reversed by 2-NP or FICZ, respectively (Fig. 5D).

### 3.6 2-NP or FICZ reversed the levels of apoptosis, T cell infiltration, and Trp-Kyn metabolism on the treatment of QFM combined with the PD-1 inhibitor

**Fig. 6:**
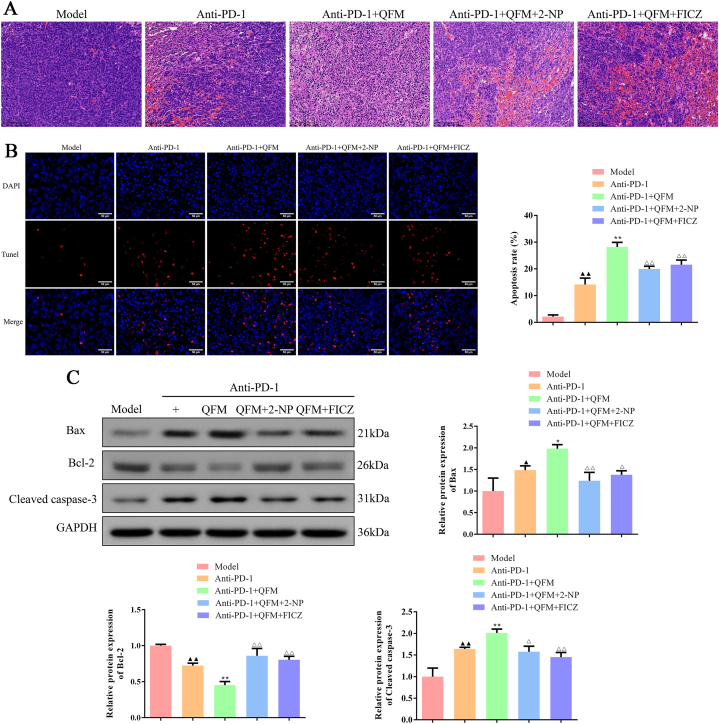
**Effect of 2-NP or FICZ on the proapoptotic role of QFM combined with a PD-1 inhibitor in LC mice *in vivo*.** (A) H&E staining analyses of tumor sections for LC mice treated with QFM and PD-1 inhibitor combined with 2-NP or FICZ (Scale bar = 100 μm). (B) TUNEL staining results in LC mice in each group (Scale bar = 50 μm). (C) The protein expression levels of Bax, Bcl-2, and Cleaved caspase-3 in tumor tissues in the lung of LC mice (The original image is provided in the Supplementary file Fig. S3). ^▲▲^*P < 0.01* compared with model group; **P<0.01 compared with anti-PD-1 group; ^△^*P < 0.05,*^△△^*P < 0.01* anti-PD-1+QFM group.

**Fig. 7:**
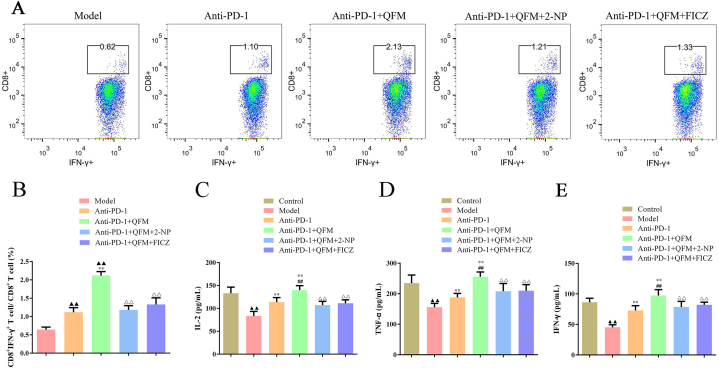
**Effect of 2-NP or FICZ on the CD8**^**+**^**IFNγ**^**+**^**T cells infiltration and immune-related factors in QFM combined with PD-1 inhibitor treated LC mice.** (A–B) Flow cytometry analysis of CD8^+^IFNγ^+^ percentage in CD8^+^ cells in each group. The levels of (C) IL-2, (D) TNF-α, and (E) IFN-γ in the serum of mice in each group were measured with commercial kits. ^▲▲^*P < 0.01* compared with control group; **P<0.01 compared with model group; ^##^*P < 0.01* anti-PD-1group; ^△△^*P < 0.01* anti-PD-1+QFM group.

**Fig. 8:**
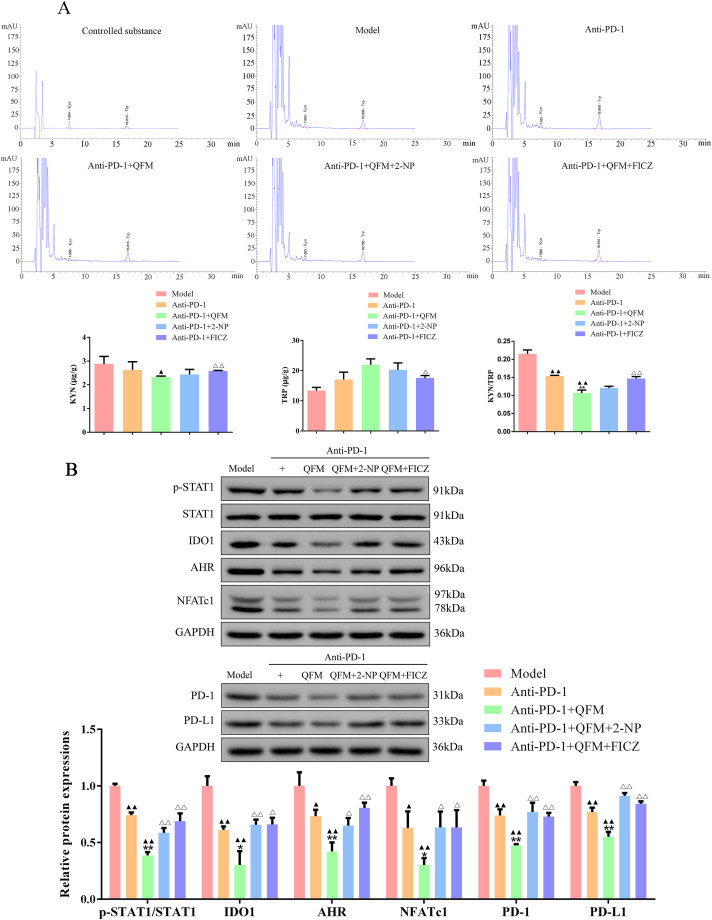
**Effect of 2-NP or FICZ on the inhibition of STAT1/IDO1-Trp-Kyn pathway in QFM combined with PD-1 inhibitor treated LC mice.** (A) HPLC analysis of Trp and Kyn contents in each group. (B) Western blot was used to measure the expression of p-STAT1, STAT1, IDO1, AhR, NFATc1, PD-1, and PD-L1 in the tumor tissues (The original image is provided in the Supplementary file Fig. S4). ^▲^*P < 0.05,*^▲▲^*P < 0.01* compared with model group; *P<0.05, **P<0.01 compared with anti-PD-1 group; ^△^*P < 0.05,*^△△^*P < 0.01* anti-PD-1+QFM group.

As illustrated in Fig. 6A, H&E staining images demonstrated that high-density cancer cells were found in the lung tumor tissues from the model group, and necrotic cancer cells were scarcely found. In contrast, the anti-PD-1 and anti-PD-1+QFM-treated groups showed much fewer tumor cell numbers compared with the model group, and clear pyknosis was observed in these groups. Afterward, a TUNEL assay was carried out to examine the apoptotic profiles of the lung tumor tissues from different groups. The outcomes indicated that the percentages of apoptotic cells were significantly elevated in the treated groups, especially the anti-PD-1+QFM-treated group (Fig. 6B). Moreover, the apoptosis-related targets were determined by Western blot, in which the protein expressions of Bax and Cleaved caspase-3 were upregulated, while Bcl-2 expression was downregulated compared to model mice (Fig. 6C; Fig. S3). Of note, the infiltration levels of CD8^+^IFNγ^+^ T cells were significantly raised in the lung tumor tissues (Fig. 7A-B). Moreover, the angiogenesis and immune regulation-related cytokines in the serum were also detected by ELISA analysis. We found the concentration of TNF-α, IL-2, and IFN-γ was markedly increased in anti-PD-1 or anti-PD-1+QFM treated groups (Fig. 7C-E). Meanwhile, a significant decrease in Kyn content and Kyn/Trp ratio and an increase in Trp content of lung tumor tissues were observed in anti-PD-1 or anti-PD-1+QFM groups compared with the model group (Fig. 8A). Similarly, Decreases in the expression of p-STAT1, IDO1, AhR, NFATc1, PD-1, and PD-L1 were found *in vivo* (Fig. 8B; Fig. S4). However, the above-mentioned improvement of anti-PD-1+QFM could be significantly attenuated by pre-treatment of 2-NP or FICZ, demonstrating that anti-PD-1+QFM could significantly prevent tumor progression in lung cancer via inhibiting the STAT1-AhR signaling axis.

## 4 Discussion

ICIs may used as promising therapeutic strategies in tumor treatment [31]. Despite the breakthroughs in the clinical application of ICIs in recent years, most patients do not benefit from ICI monotherapy [32]. As a result, there is a pressing requirement to identify approaches for enhancing the effectiveness of ICIs in clinical practice. Trp is an essential amino acid in the body, which is not only involved in protein synthesis but also a precursor for the synthesis of many microbial and host metabolites [33]. Disorders of Trp metabolism could cause apoptosis and dysfunction of immune cells, favoring the formation of an immunosuppressive microenvironment, which in turn affects the efficacy of ICIs [34]. In addition, Trp metabolism has the Kyn pathway, 5-hydroxytryptamine (5-HT) pathway, and indole pathway, of which the most important is the Kyn pathway, which plays a central function in immunological escape [33]. In this paper, we found that QFM administration might strengthen the anti-malignancy response of PD-1 inhibitor *in vivo*, as illustrated by an increase of TNF-α, IFN-γ, Trp levels, and infiltration of CD8^+^ T cells, along with a decrease of Kyn levels and PD-1 expressions in tumor tissues, which also suggested that the anti-tumorigenesis effect of QFM was mediated mainly through the PD-1 in CD8^+^ T cells and Trp-Kyn pathway.

IDO1 is the first rate-limiting enzyme in tryptophan catabolism, which can induce apoptosis or dysfunction of T cells and NK cells by mediating the depletion of tryptophan and the accumulation of its metabolite kynurenine, thus weakening the body's anti-tumor immunity [35,36]. Meanwhile, as promising immunotherapeutic candidates, IDO1 inhibitors, such as epacadostat and indoximod, have already entered clinical trials [37,38]. In particular, the combination of IDO1 inhibitors with PD-1 antibodies could drastically enhance the objective response rate of tumor therapy [39]. Therefore, the selection of IDO1 inhibitors that contribute to activating T and NK cells to break the immune tolerance of tumors may bring breakthroughs in tumor immunotherapy. In the current study, QFM combined with PD-1 inhibitor inhibited the phosphorylation of STAT1 and the protein expression levels of IDO1 in lung tumor tissues *in vivo*. Additionally, the QFM-containing serum not only reduced the cell viability and Kyn content but also promoted cell apoptosis of IFN-γ-mediated A549 and LLC-Luc cells. Our experimental findings demonstrated that the decrease in phosphorylation of STAT1 and the protein expression levels of IDO1 are closely associated with suppression of proliferation and induction of apoptosis in NSCLC cells.

STAT1, as an important transcription factor for IFN-γ-induced IDO1 expression, is activated by JAk kinase in the cytoplasmic tail region of the IFN-γ receptor, and links to the GAS pattern to directly activate the transcriptional expression of IDO1 [40,41]. On the other hand, AhR is a subfamily member of the basic helix-loop-helix (HLH)-PAS superfamily, a ligand-activated transcription factor [42]. More importantly, kynurenine is an agonist of AHR [42,43]. To explore the anti-tumor principles of QFM combined with PD-1 inhibitor in the LLC-luc tumor-bearing C57BL/6 mice, it is the first time that the 2-NP and FICZ have been administrated with QFM and PD-1 inhibitor in our study. Interestingly, PD-1 inhibitor and QFM used alone or in combination showed significant changes in the tumor growth, thymus index, spleen index, tumor tissue necrosis and apoptosis, Trp-Kyn metabolism, and STAT1-AhR signaling axis in LLC-luc tumor-bearing C57BL/6 mice.

Accumulating data also indicated that CD8^+^ T cells in tumor tissues are activated by antigenic stimulation, and IL-2 in the tumor microenvironment promotes their differentiation into cytotoxic T lymphocytes (CTLs) and infiltration into the tumor microenvironment [44,45]. The CTLs can either directly induce apoptosis of the tumor cells, or produce IFN-γ to kill the tumor cells. These CTLs contain IFN-γ, which can be detected by combining with IFN-γ flow antibody, i.e., CD8^+^IFNγ^+^ T cells; in turn, IFN-γ can increase the number of CTL cells and make them produce more IFN-γ by promoting CD8^+^ T phenotypic differentiation. Besides, PD-1 antibody binds to PD-L1 receptors on the surface of T cells, which can release T cell immunosuppression [46,47]. The results of the current study showed that QFM combined with PD-1 inhibitor improved CD8^+^ T-cell status, increased the proportion of IFN-γ^+^ cells and the content of IL-2, TNF-α and IFN-γ in the serum samples of LLC-luc tumor-bearing C57BL/6 mice, indicating that QFM combined with PD-1 inhibitor could promote the infiltration of CD8^+^ T-cells and the release of IFN-γ, thus suppressing the tumor. However, the anti-proliferative effect of QFM combined with PD-1 inhibitor was significantly attenuated through 2-NP or FICZ treatment. Generally, suppressing the STAT1/IDO1-mediated tryptophan-kynurenine pathway is regarded as an important strategy of QFM combined with PD-1 inhibitors against lung cancer. However, we did not do additional in-depth investigation of the clinical practice; which is the essential limitation that needs to be addressed in our subsequent research.

## 5 Conclusion

In conclusion, the anti-cancer effects of QFM combined with PD-1 inhibitor are mainly through inhibiting activation of STAT1/IDO1-mediated tryptophan-kynurenine pathway and down-regulating expression of PD-1. These findings suggest that QFM combined with PD-1 inhibitor can be used as an alternate method for treating lung cancer via reprogramming tryptophan-kynurenine axis metabolism.

## Funding

This work was supported by the 10.13039/501100001809National Natural Science Foundation of China (No.82374535), 10.13039/501100004731Zhejiang Provincial Natural Science Foundation Project (Exploring Youth) (No. Q24H290030), and General Scientific Research Projects of the Department of Education Zhejiang (NO·Y202351357).

## Ethics approval

Animal care and experimental procedures were approved by the Animal Experimentation Ethics Committee of Zhejiang Eyong Pharmaceutical Research and Development Center (No. ZJEY-20221024-03).

## Data availability statement

The datasets used and/or analyzed during the current study are available from the corresponding author upon reasonable request.

## CRediT authorship contribution statement

**Zhuo Chen:** Writing – original draft, Visualization, Investigation, Data curation. **Yu-Heng Ding:** Writing – review & editing, Investigation. **Lan Shao:** Writing – review & editing, Investigation. **Xu-Ming Ji:** Writing – review & editing, Methodology, Conceptualization. **Xiang Qian:** Writing – review & editing, Investigation. **Ai-Qin Zhang:** Writing – review & editing, Supervision, Funding acquisition, Conceptualization.

## Declaration of competing interest

The authors declare that they have no known competing financial interests or personal relationships that could have appeared to influence the work reported in this paper.
