# Supplementary material for: Qingfei mixture mitigates immunosuppression of tumor microenvironment in non-small cell lung cancer by blocking stat1/Ido1-mediated tryptophan-kynurenine pathway
Source: Heliyon. 2024 May 31;10(11):e32260. doi: 10.1016/j.heliyon.2024.e32260 (PMC11176930; doi:10.1016/j.heliyon.2024.e32260)
Supplement: Multimedia component 1 [file mmc1.pdf]

Original uncropped images of western blots for the Figure 1D

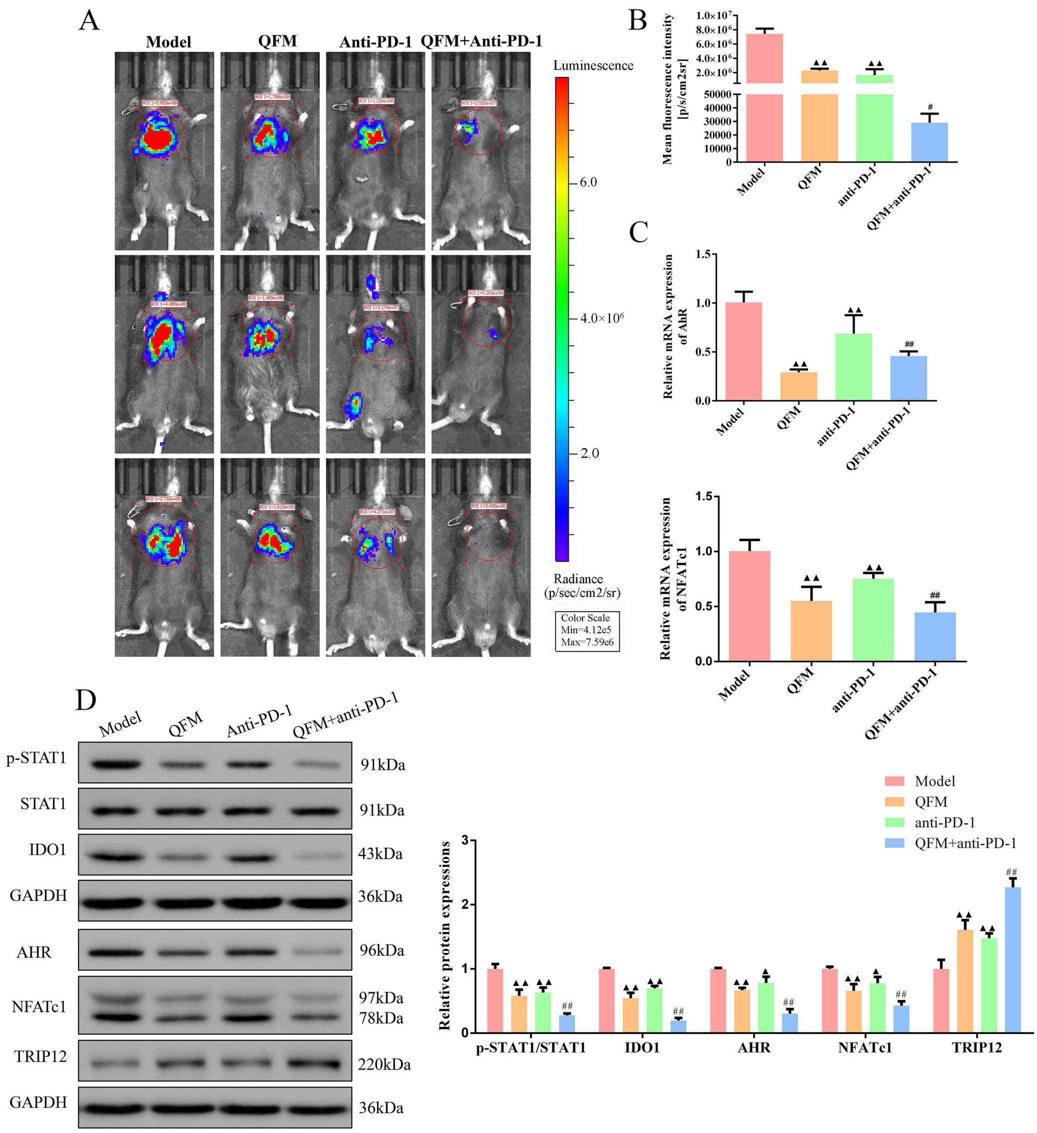

Figure 1 Effect of QFM combined with PD-1 inhibitor on tumor growth in LC mice *in vivo* by targeting STAT1/IDO1-Trp-Kyn pathway. (A) Representative images with fluorescence signal in living LC mice. (B) The mean of fluorescence signal in LC mice. (C) The mRNA level of AhR and NFATc1 in tumor tissues of LC mice using quantitative RT-PCR analysis. (D) Western blot measured the expression of p-STAT1, STAT1, IDO1, AhR, NFATc1, and TRIP12 in tumor tissues of LC mice. $^{\blacktriangle}$*P<0.05*, $^{\blacktriangle\blacktriangle}$*P<0.01* compared with model group; $^{\#}$*P<0.05*, $^{\#\#}$*P<0.01* compared with anti-PD-1 group.

<table>
<tr><th>Genes</th><th></th></tr>
<tr><td>p-STAT1</td><td>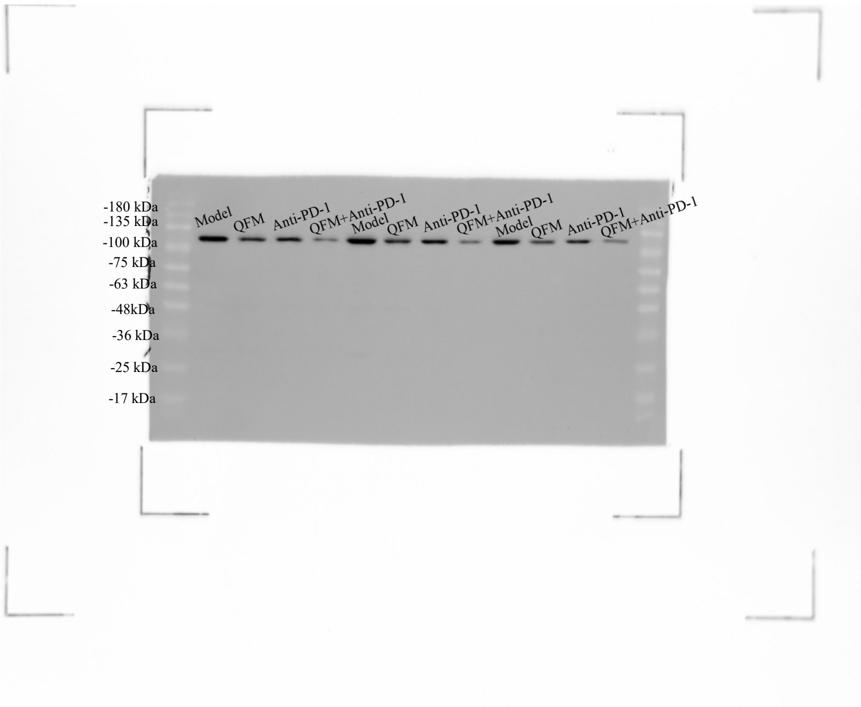
</td></tr>
<tr><td>STAT1</td><td>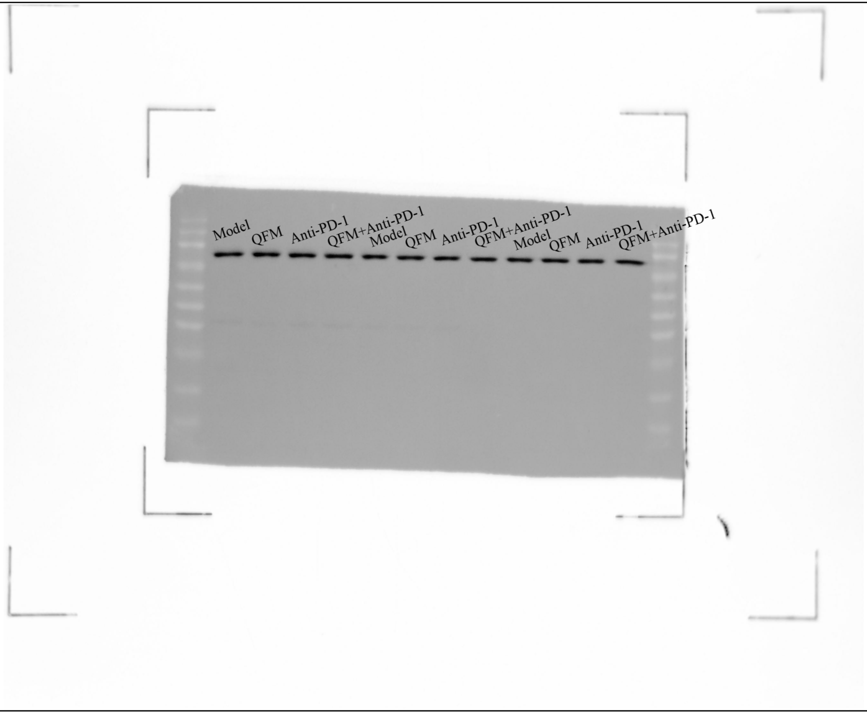
</td></tr>
</table>

<table>
<tr><td>IDO1</td><td>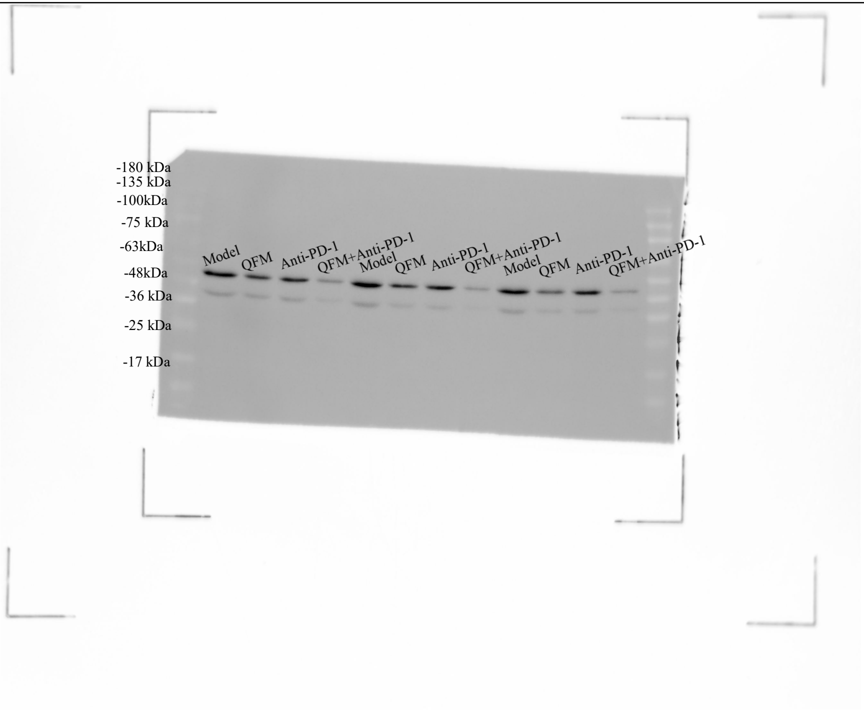
</td></tr>
<tr><td>GAPDH</td><td>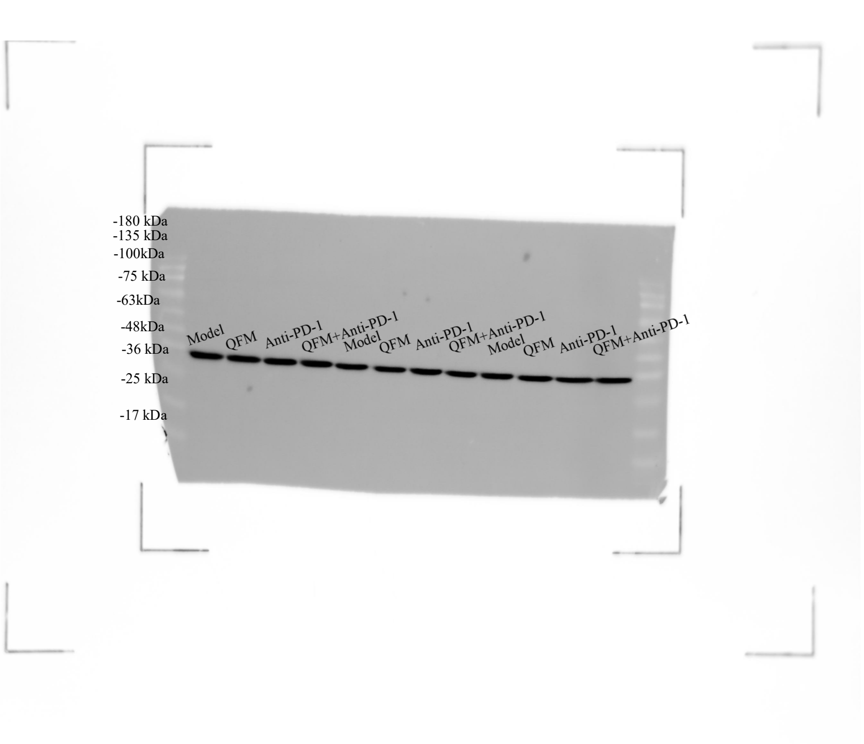
</td></tr>
</table>

<table>
<tr>
<td>AHR</td>
<td>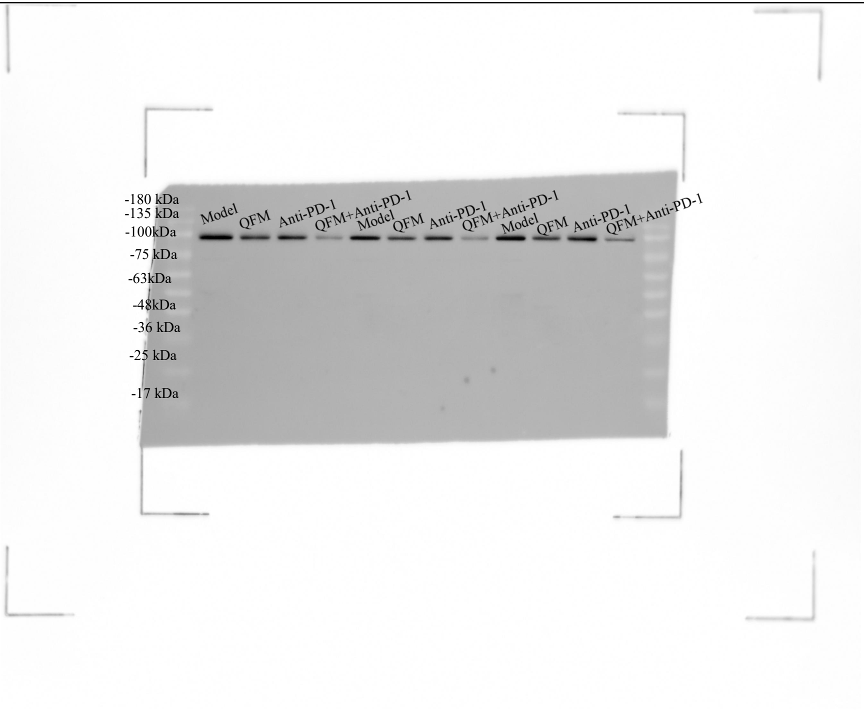
</td>
</tr>
<tr>
<td>NFATc1</td>
<td>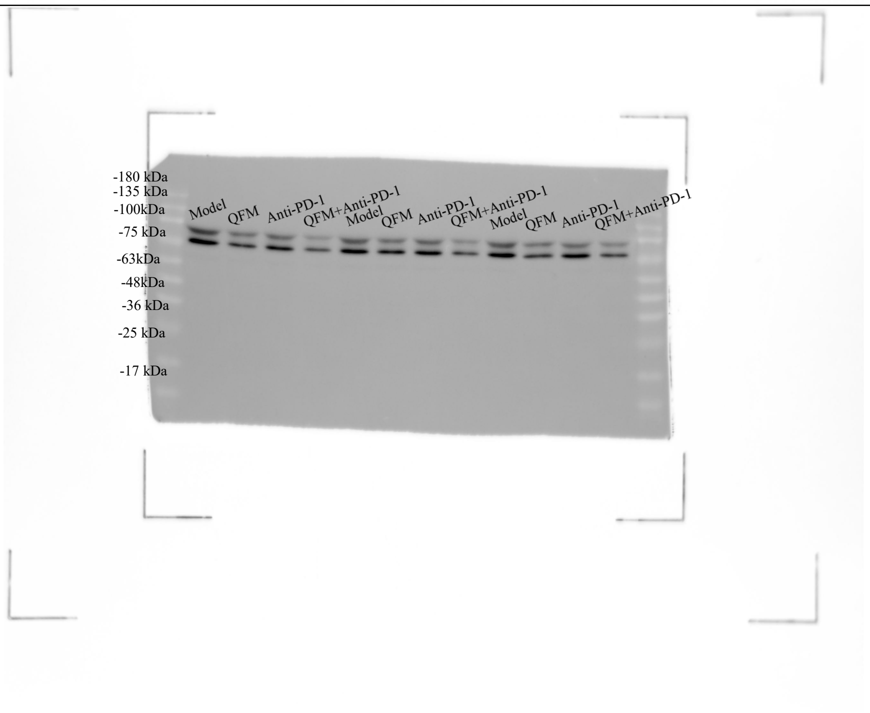
</td>
</tr>
</table>

<table>
<tr><td>TRIP12</td><td>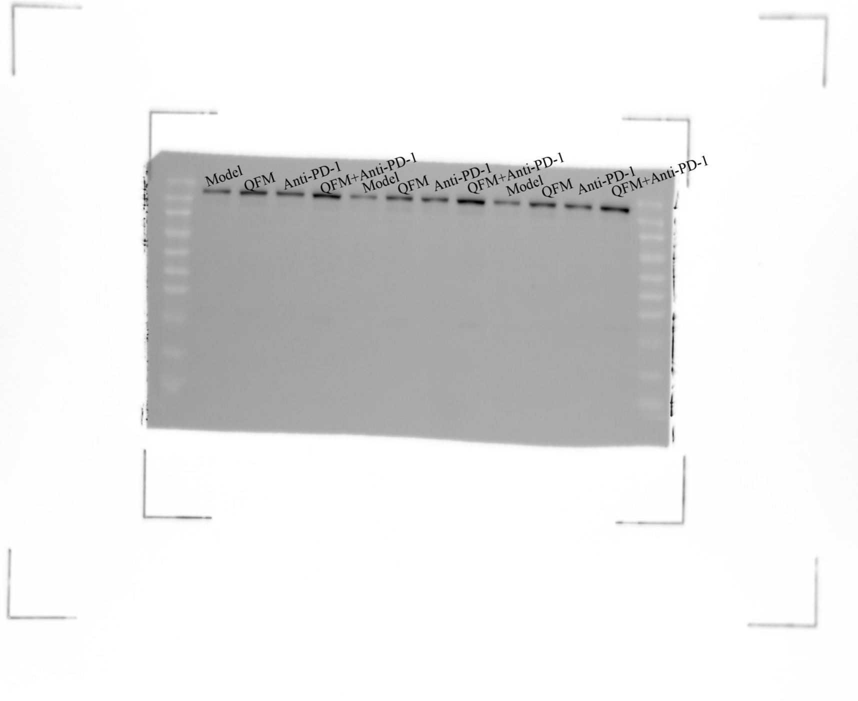
</td></tr>
<tr><td>GAPDH</td><td>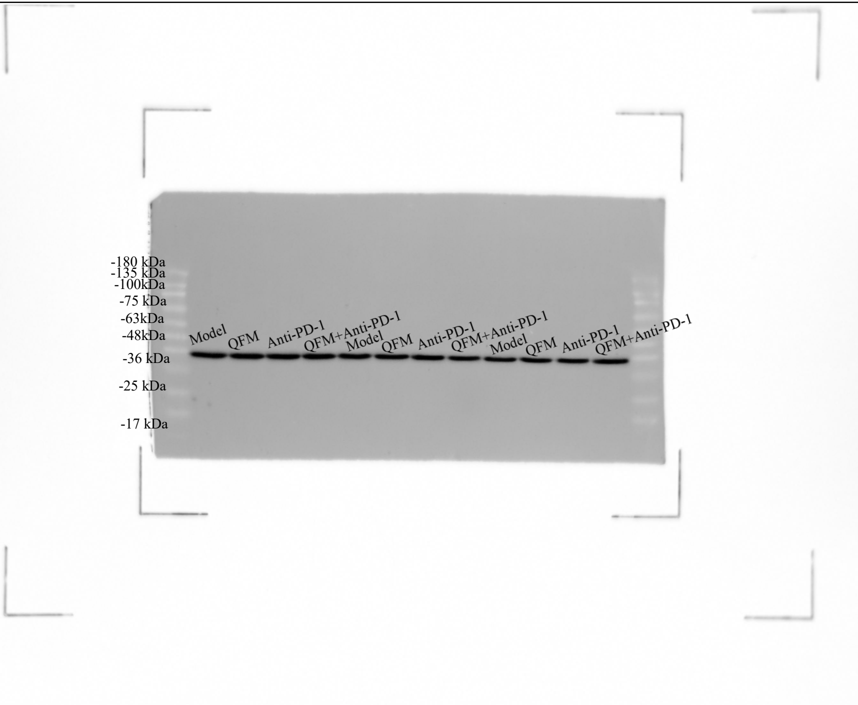
</td></tr>
</table>
