# Supplementary material for: Qingfei mixture mitigates immunosuppression of tumor microenvironment in non-small cell lung cancer by blocking stat1/Ido1-mediated tryptophan-kynurenine pathway
Source: Heliyon. 2024 May 31;10(11):e32260. doi: 10.1016/j.heliyon.2024.e32260 (PMC11176930; doi:10.1016/j.heliyon.2024.e32260)
Supplement: Multimedia component 2 [file mmc2.pdf]

Original uncropped images of western blots for the Figure 4D

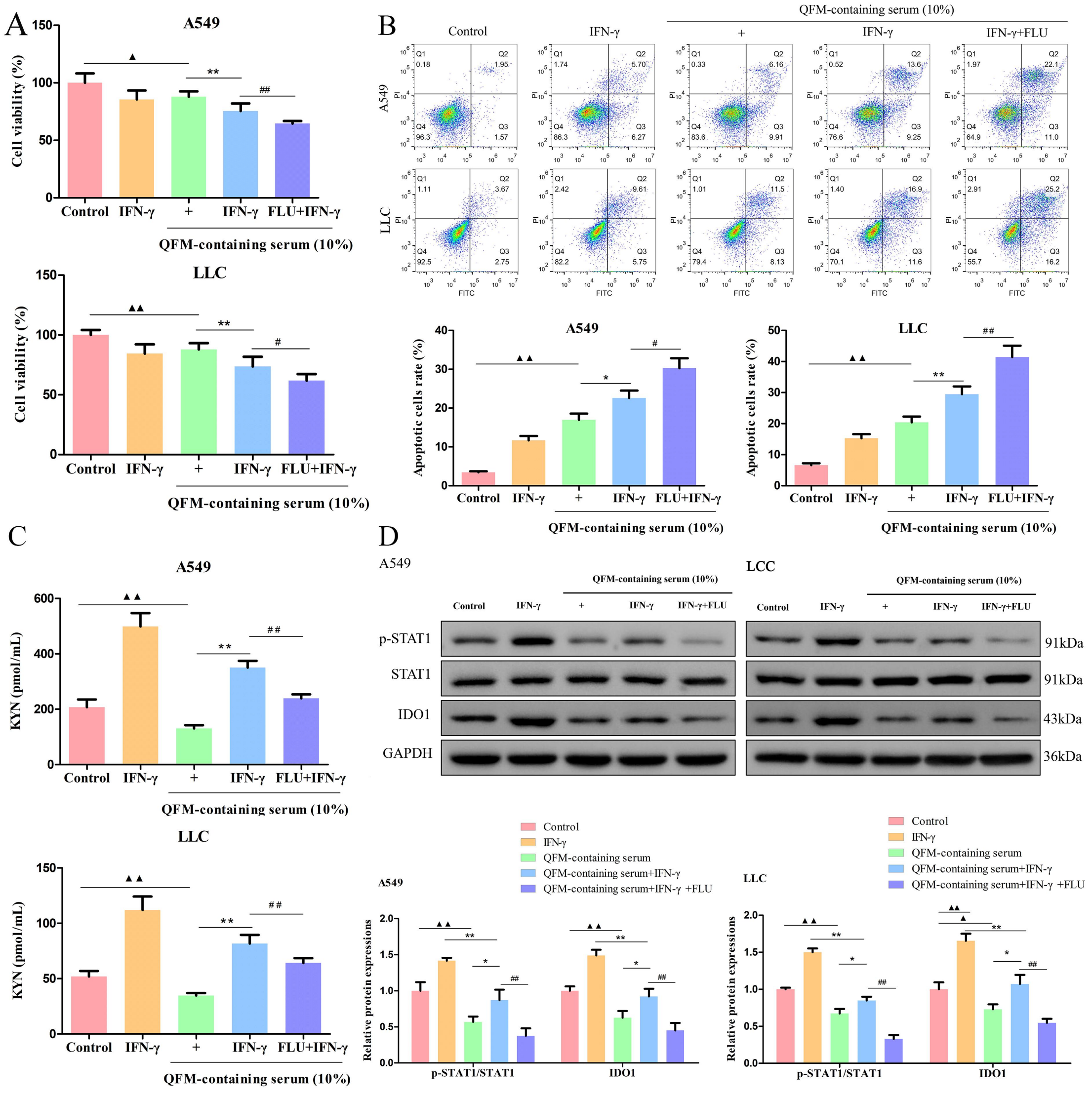

Figure 4 Effect of QFM-containing serum on STAT1 to inhibit IDO1-Trp-Kyn signaling pathway *in vitro*. (A) Cell Counting Kit-8 (CCK-8) was used to detect the cell viability of IFN-γ-mediated A549 and LLC cells treated with QFM-containing serum or in combination with FLU (5 μM, STAT1 inhibitor). (B) Flow cytometric analysis on the cell apoptosis in IFN-γ-mediated A549 and LLC cells. (C) ELISA for detecting Kyn contents in IFN-γ-mediated A549 and LLC cells. (D) Western blotting for detecting p-STAT1, STAT1, and IDO1 expression levels in IFN-γ-mediated A549 and LLC cells. ▲*P<0.05,* ▲▲*P<0.01* compared with control group; **P<0.05,* ***P<0.01* compared with QFM-containing serum group; #*P<0.05,* ##*P<0.01* compared with QFM-containing serum+IFN-γ group.

<table>
<tr><th>Genes</th><th colspan="3">A549</th></tr>
<tr><td rowspan="4">p-STAT1</td><td>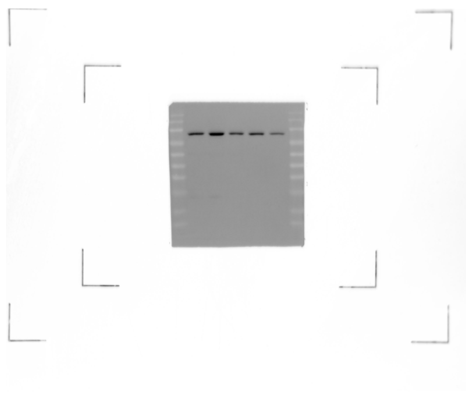</td><td>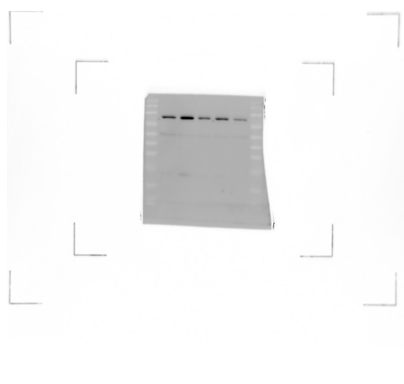</td><td>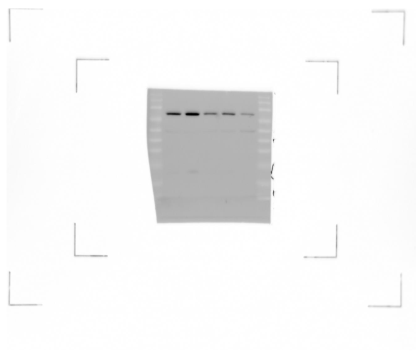</td></tr>
<tr><th colspan="3">LCC</th></tr>
<tr><td>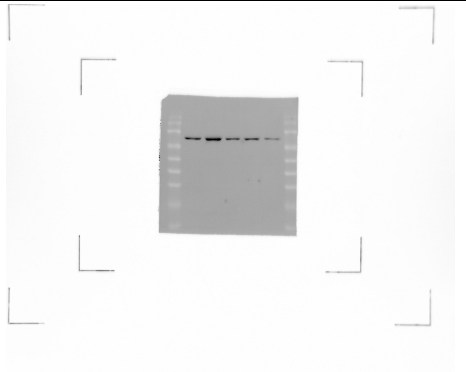</td><td>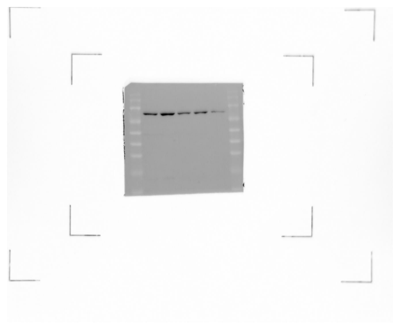</td><td>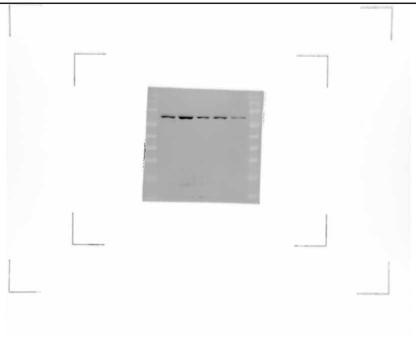</td></tr>
<tr></tr>
<tr><td rowspan="4">STAT1</td><th colspan="3">A549</th></tr>
<tr><td>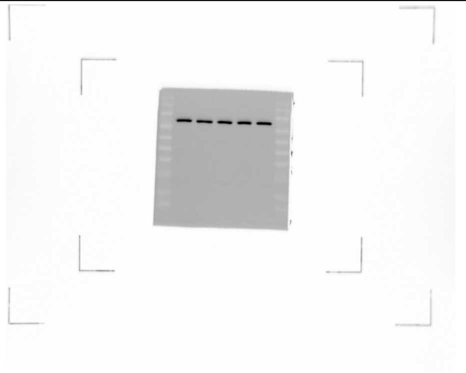</td><td>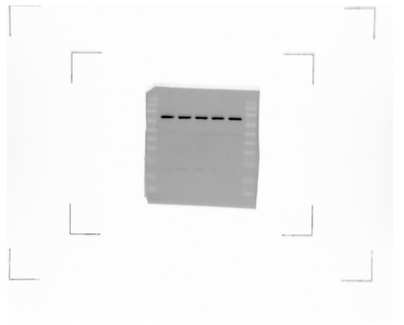</td><td>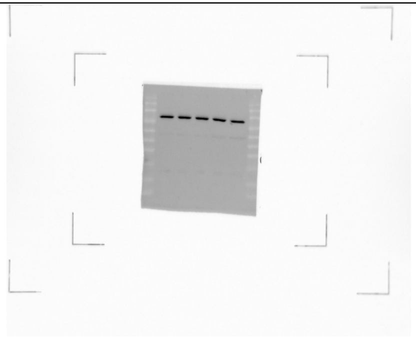</td></tr>
<tr><th colspan="3">LCC</th></tr>
<tr><td>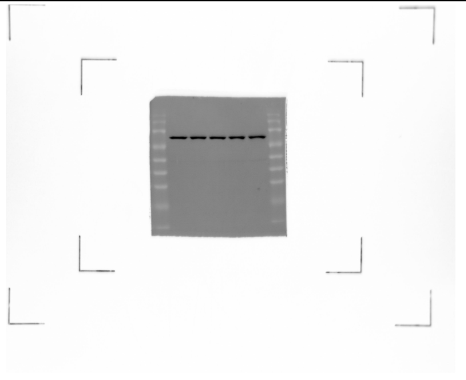</td><td>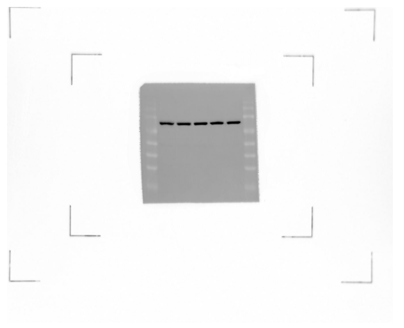</td><td>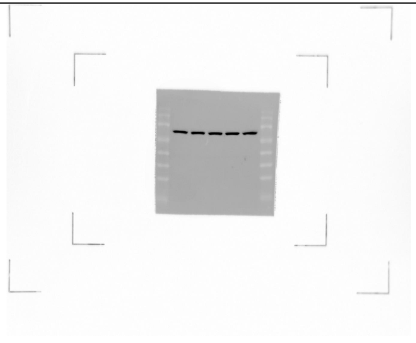</td></tr>
<tr><td></td><td colspan="3"></td></tr>
</table>

<table>
<tr><td rowspan="4">IDO1</td><td colspan="3">A549</td></tr>
<tr><td>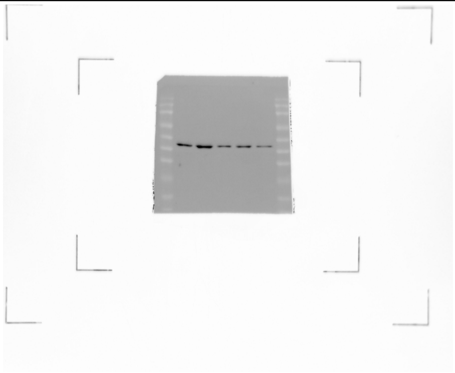</td><td>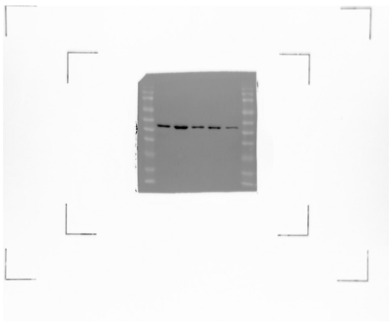</td><td>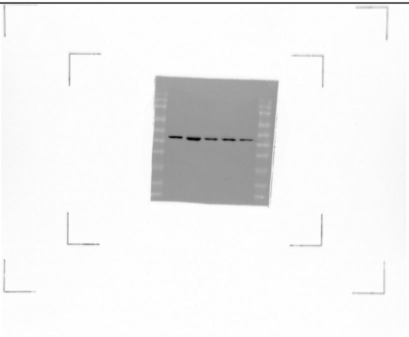</td></tr>
<tr><td colspan="3">LCC</td></tr>
<tr><td>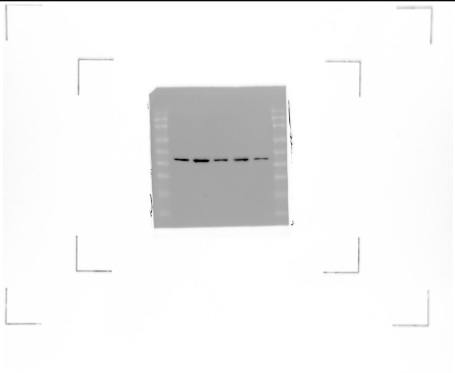</td><td>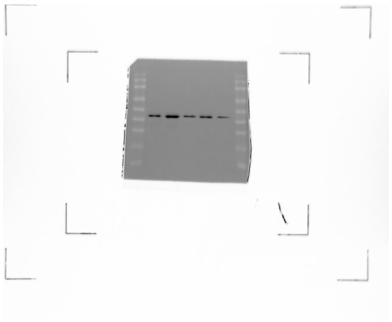</td><td>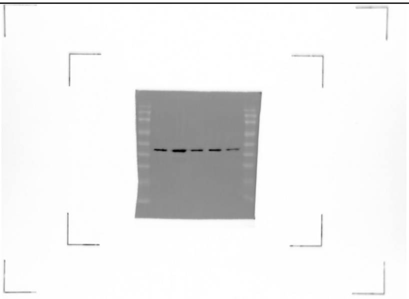</td></tr>
<tr><td rowspan="4">GAPDH</td><td colspan="3">A549</td></tr>
<tr><td>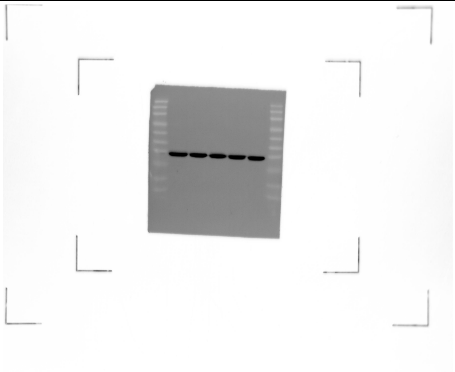</td><td>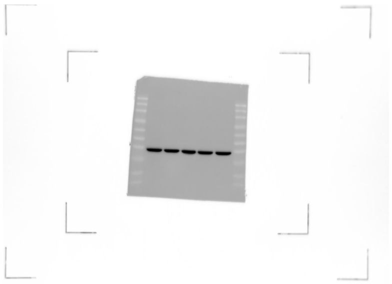</td><td>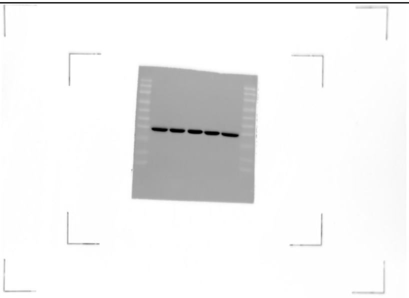</td></tr>
<tr><td colspan="3">LCC</td></tr>
<tr><td>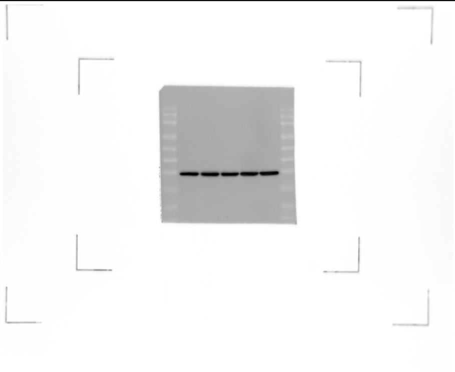</td><td>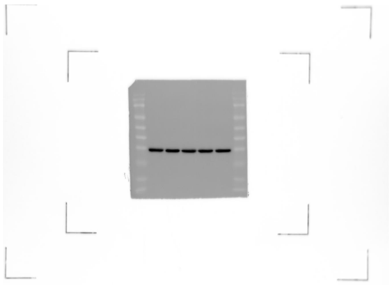</td><td>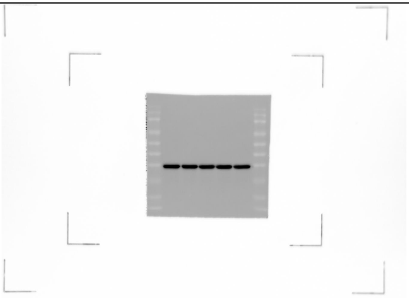</td></tr>
</table>
