# Supplementary material for: Qingfei mixture mitigates immunosuppression of tumor microenvironment in non-small cell lung cancer by blocking stat1/Ido1-mediated tryptophan-kynurenine pathway
Source: Heliyon. 2024 May 31;10(11):e32260. doi: 10.1016/j.heliyon.2024.e32260 (PMC11176930; doi:10.1016/j.heliyon.2024.e32260)
Supplement: Multimedia component 3 [file mmc3.pdf]

Original uncropped images of western blots for the Figure 6C

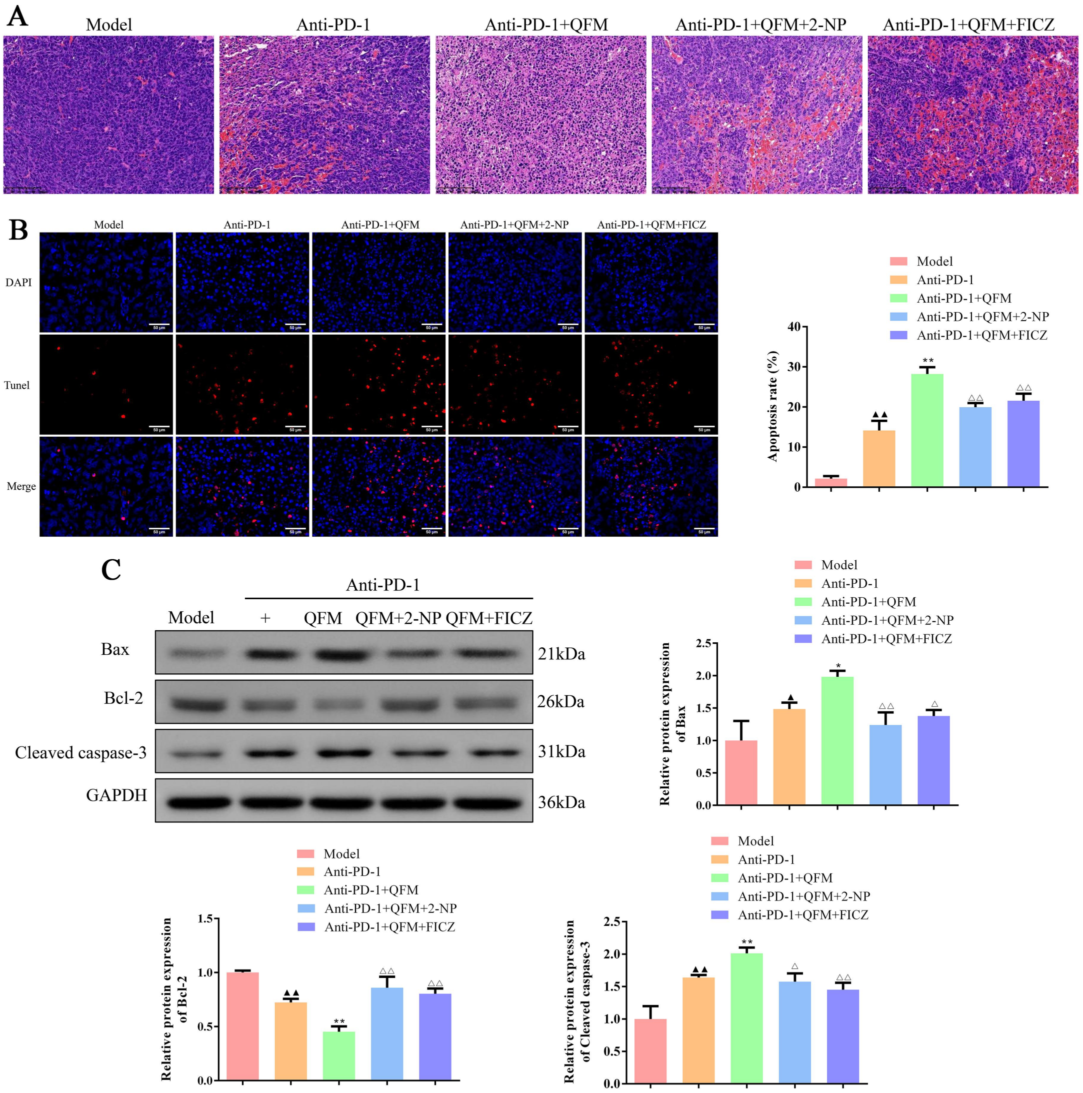

Figure 6 Effect of 2-NP or FICZ on the proapoptotic role of QFM combined with a PD-1 inhibitor in LC mice *in vivo*. (A) H&E staining analyses of tumor sections for LC mice treated with QFM and PD-1 inhibitor combined with 2-NP or FICZ (Scale bar = 100 μm). (B) TUNEL staining results in LC mice in each group (Scale bar = 50 μm). (C) The protein expression levels of Bax, Bcl-2, and Cleaved caspase-3 in tumor tissues in the lung of LC mice. ▲▲*P<0.01* compared with model group; ***P<0.01* compared with anti-PD-1 group; △*P<0.05,* △△*P<0.01* anti-PD-1+QFM group.

**Genes**			
Bax	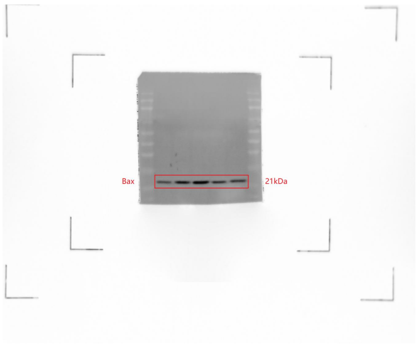	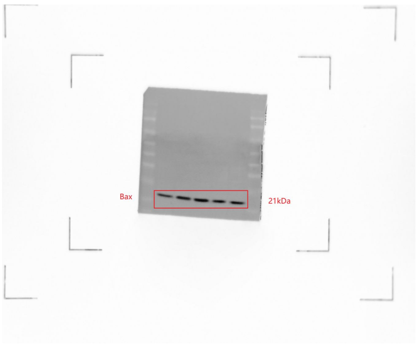	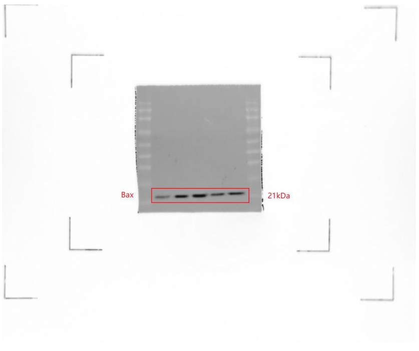
Bcl-2	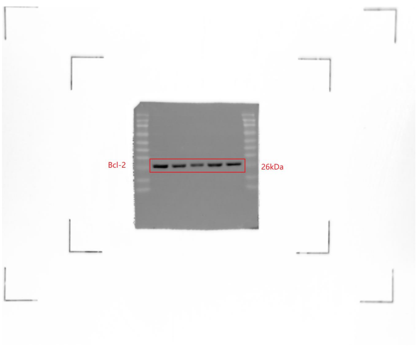	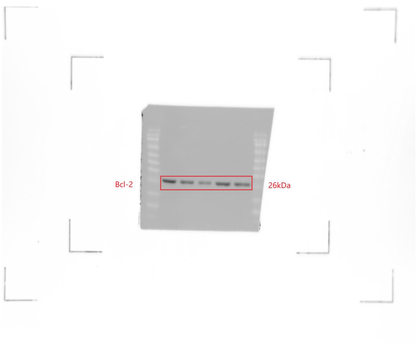	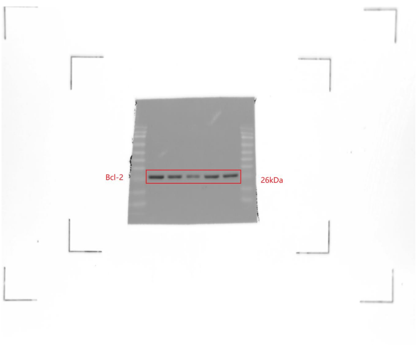
Cleaved caspase-3	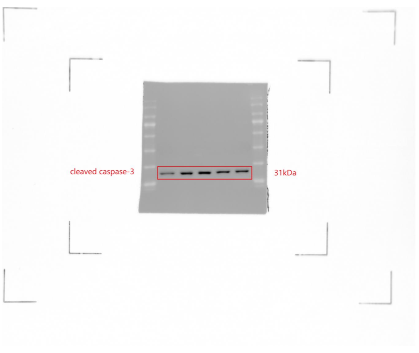	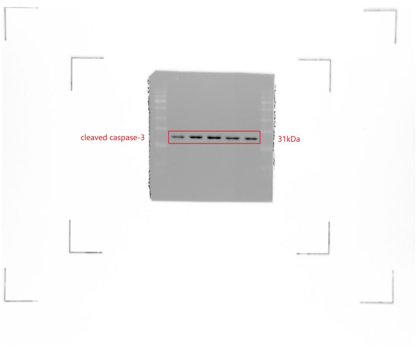	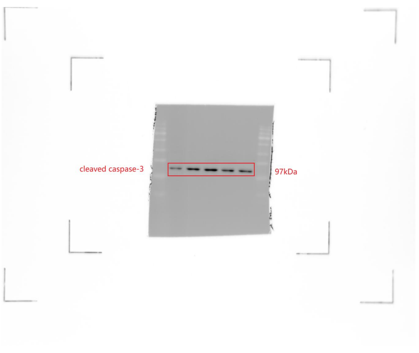
GAPDH	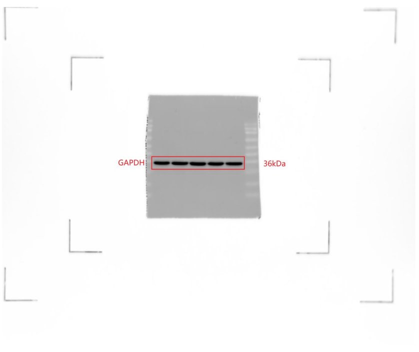	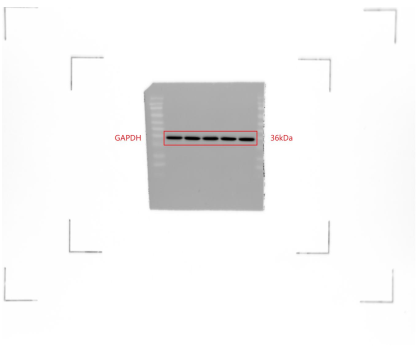	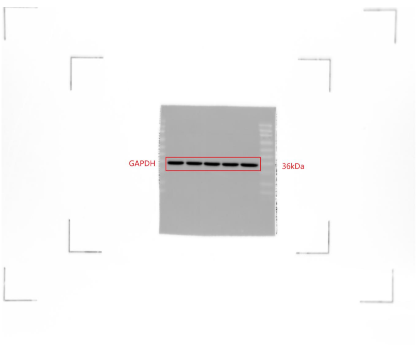
