# Supplementary material for: Qingfei mixture mitigates immunosuppression of tumor microenvironment in non-small cell lung cancer by blocking stat1/Ido1-mediated tryptophan-kynurenine pathway
Source: Heliyon. 2024 May 31;10(11):e32260. doi: 10.1016/j.heliyon.2024.e32260 (PMC11176930; doi:10.1016/j.heliyon.2024.e32260)
Supplement: Multimedia component 4 [file mmc4.pdf]

Original uncropped images of western blots for the Figure 8B

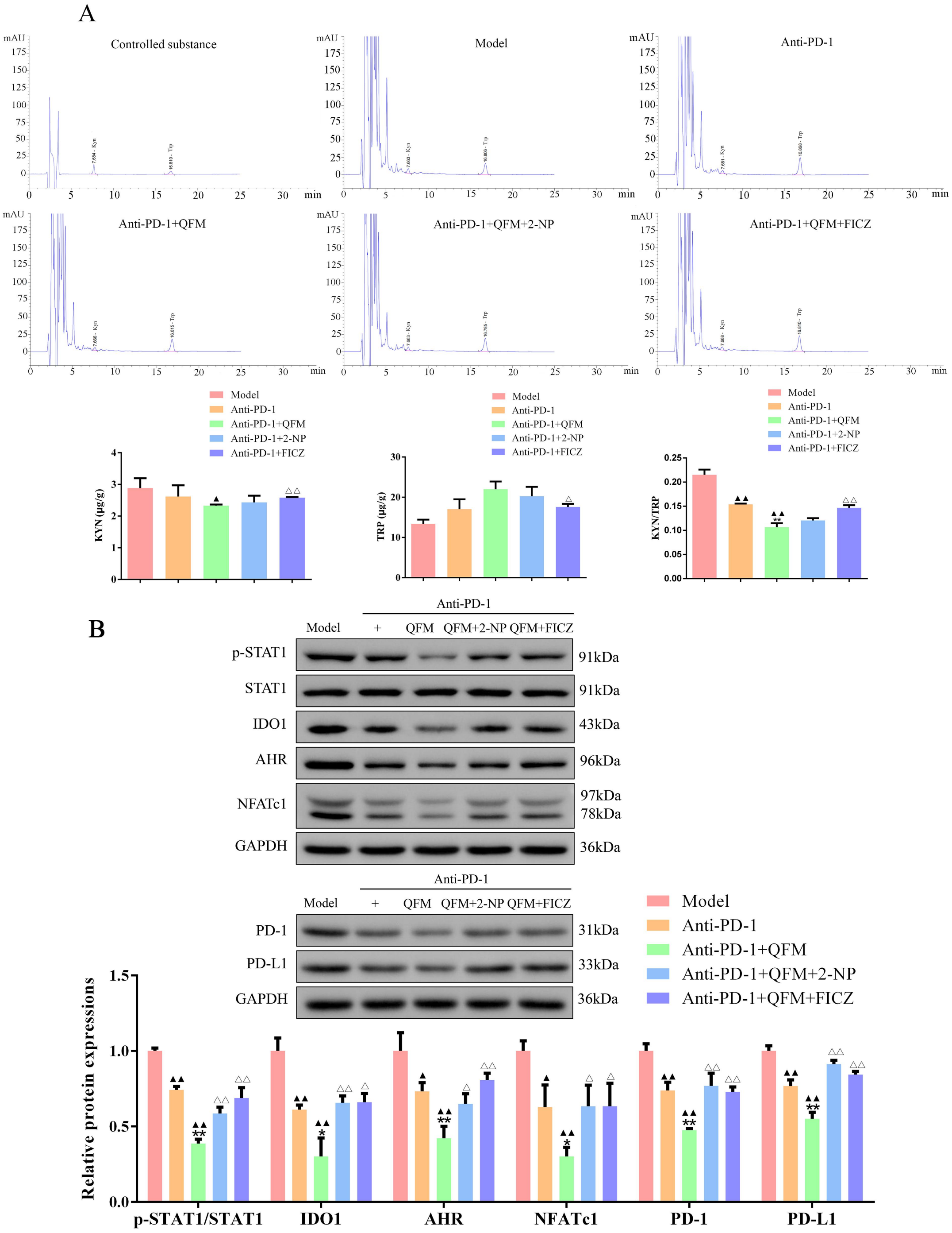

Figure 8 Effect of 2-NP or FICZ on the inhibition of STAT1/IDO1-Trp-Kyn pathway in QFM combined with PD-1 inhibitor treated LC mice. (A) HPLC analysis of Trp and Kyn contents in each group. (B) Western blot was used to measure the expression of p-STAT1, STAT1, IDO1, AhR, NFATc1, PD-1, and PD-L1 in the tumor tissues. ▲*P<0.05,* ▲▲*P<0.01* compared with model group; **P<0.05,* ***P<0.01* compared with anti-PD-1 group; △*P<0.05,* △△*P<0.01* anti-PD-1+QFM group.

**Genes**			
p-STAT1	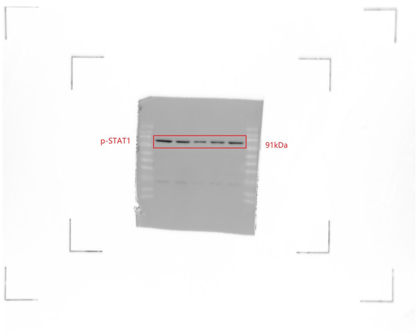	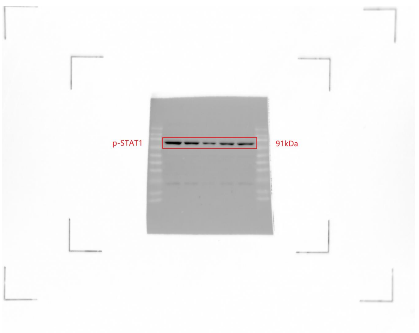	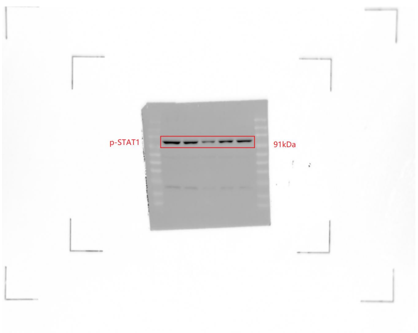
STAT1	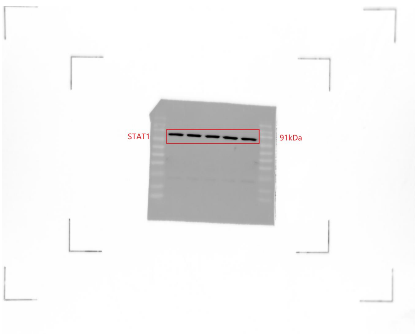	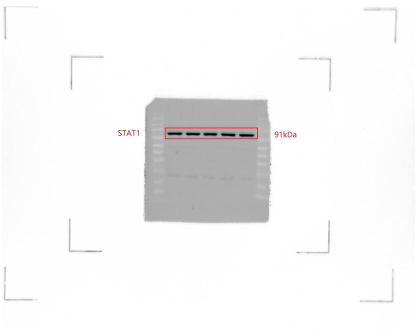	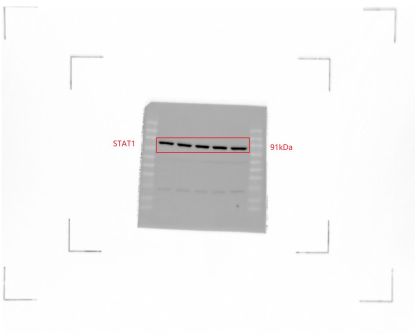
IDO1	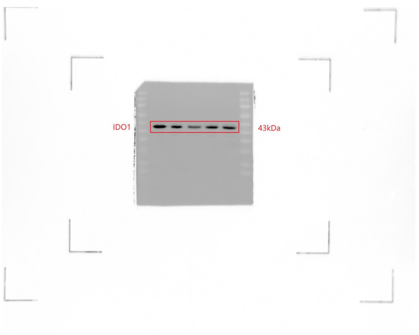	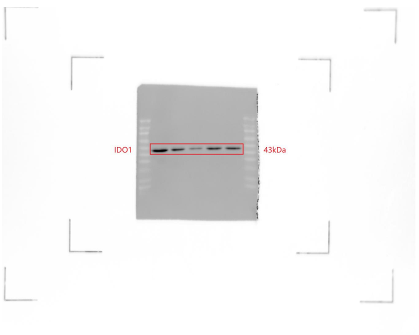	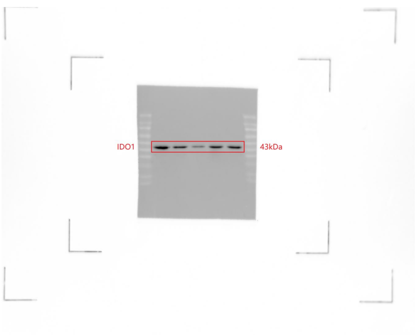
AHR	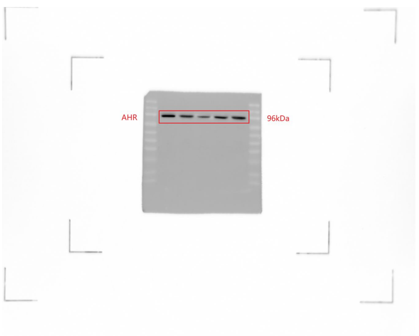	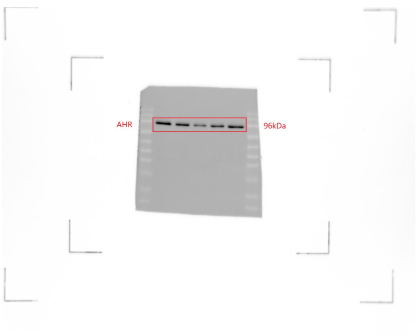	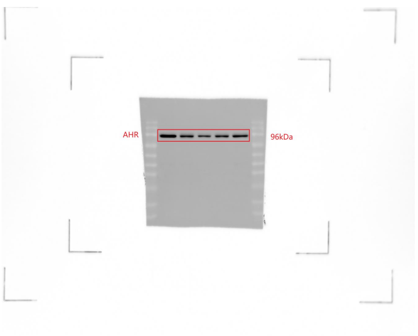

NFATc1	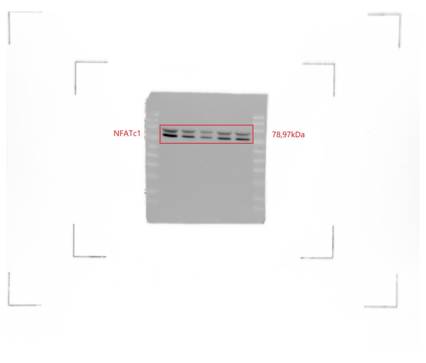	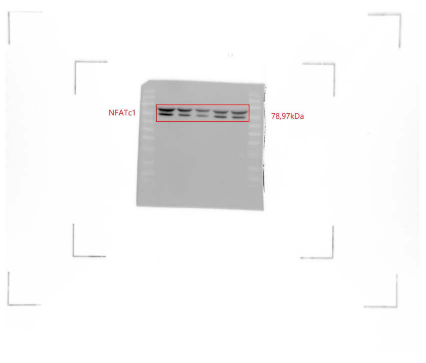	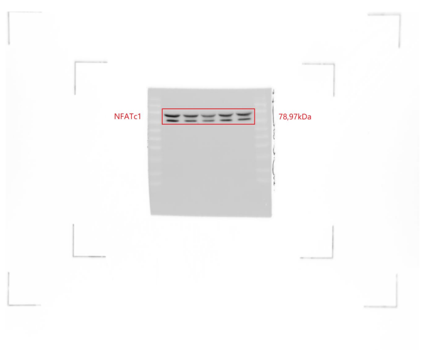
GAPDH	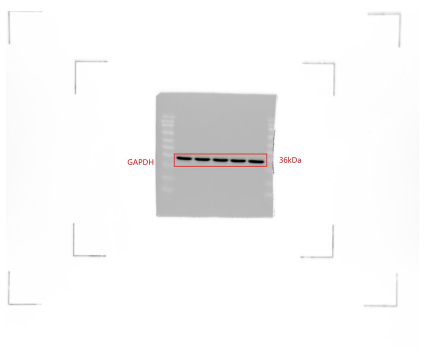	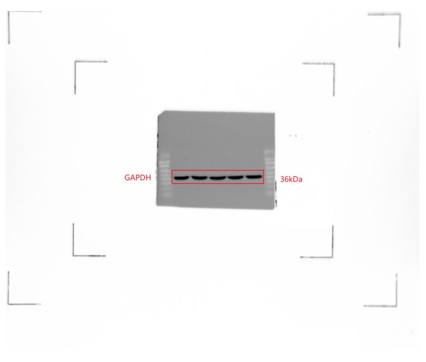	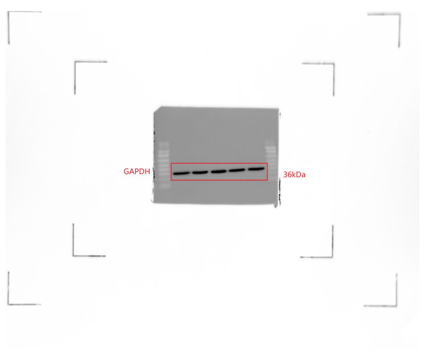
PD-1	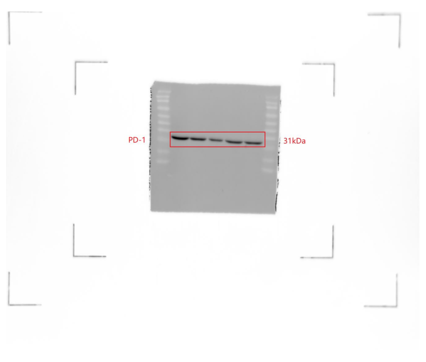	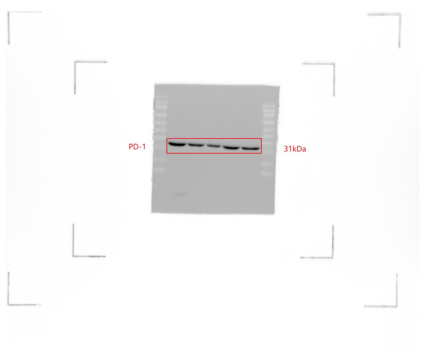	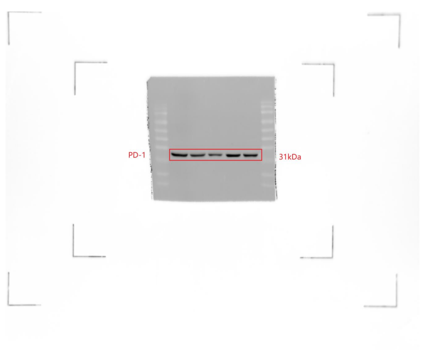
PD-L1	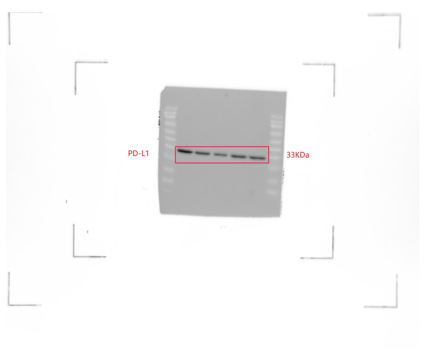	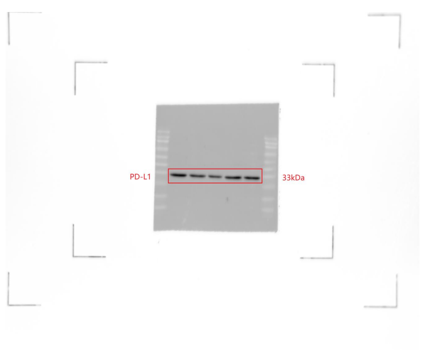	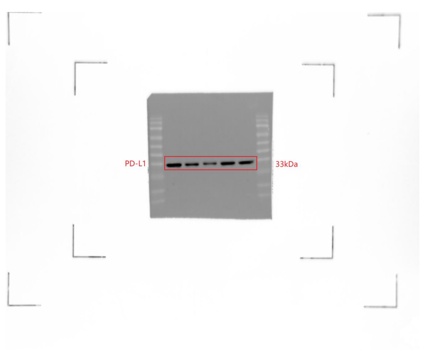

GAPDH	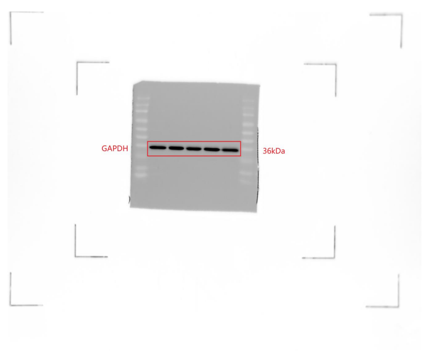	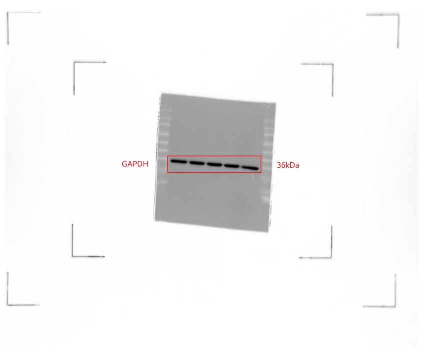	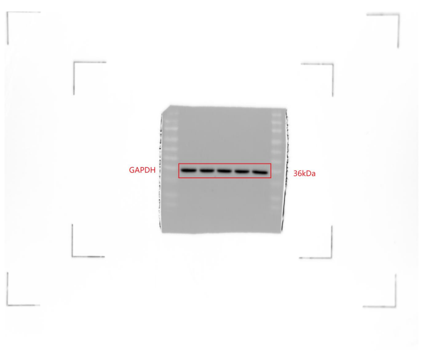
